# TRIM50 inhibits glycolysis and the malignant progression of gastric cancer by ubiquitinating PGK1

**DOI:** 10.7150/ijbs.97091

**Published:** 2024-07-01

**Authors:** Chao Gu, Yiwen Xia, Chen Lu, Shengkui Qiu, Jihuan Wang, Lu Zhang, Jialun Lv, Tianlu Jiang, Lang Fang, Penghui Xu, Zetian Chen, Ying Li, Li Xie, Zekuan Xu, Bowen Li

**Affiliations:** 1 Department of General Surgery, The Affiliated Suzhou Hospital of Nanjing Medical University, Suzhou, 215000, Jiangsu Province, China.; 2 Department of General Surgery, The First Affiliated Hospital of Nanjing Medical University, Nanjing, 210029, Jiangsu Province, China.; 3 Department of General Surgery, The Second Affiliated Hospital of Nanjing Medical University, Nanjing, 210011, Jiangsu Province, China.; 4 Department of General Surgery, The Second Affiliated Hospital of Nantong University, Nantong, 226001, Jiangsu Province, China.; 5 Department of General Surgery, Affiliated People's Hospital of Jiangsu University, Zhenjiang, 212000, Jiangsu Province, China.; 6 Jiangsu Key Lab of Cancer Biomarkers, Prevention and Treatment, Collaborative Innovation Center for Personalized Cancer Medicine, Nanjing Medical University, Nanjing, 211166, Jiangsu Province, China.

**Keywords:** Gastric cancer, ubiquitination, glycolysis, tumor-associated macrophages, m6A

## Abstract

Ubiquitination plays a pivotal regulatory role in tumor progression. Among the components of the ubiquitin-proteasome system (UPS), ubiquitin-protein ligase E3 has emerged as a key molecule. Nevertheless, the biological functions of E3 ubiquitin ligases and their potential mechanisms orchestrating glycolysis in gastric cancer (GC) remain to be elucidated. In this study, we conducted a comprehensive transcriptomic analysis to identify the core E3 ubiquitin ligases in GC, followed by extensive validation of the expression patterns and clinical significance of Tripartite motif-containing 50 (TRIM50) both *in vitro* and *in vivo*. Remarkably, we found that TRIM50 was downregulated in GC tissues, associated with malignant progression and poor patient survival. Functionally, overexpression of TRIM50 suppressed GC cell proliferation and indirectly mitigated the invasion and migration of GC cells by inhibiting the M2 polarization of tumor-associated macrophages (TAMs). Mechanistically, TRIM50 inhibited the glycolytic pathway by ubiquitinating Phosphoglycerate Kinase 1 (PGK1), thereby directly suppressing GC cell proliferation. Simultaneously, the reduction in lactate led to diminished M2 polarization of TAMs, indirectly inhibiting the invasion and migration of GC cells. Notably, the downregulation of TRIM50 in GC was mediated by the METTL3/YTHDF2 axis in an m6A-dependent manner. In our study, we definitively identified TRIM50 as a tumor suppressor gene (TSG) that effectively inhibits glycolysis and the malignant progression of GC by ubiquitinating PGK1, thus offering novel insights and promising targets for the diagnosis and treatment of GC.

## Introduction

Gastric cancer (GC) is one of the most common malignancies worldwide. Despite advancements in comprehensive treatment approaches for GC, the overall 5-year survival rate of patients remains low 1. Molecular targeted therapies have achieved success in the treatment of various cancers; however, effective targeted drugs for GC are still lacking 2. Therefore, it is imperative to delve into the molecular mechanisms driving GC development and identify optimized therapeutic strategies to address treatment challenges.

Ubiquitination is a pivotal cellular regulatory mechanism involved in the modulation of cellular proliferation, survival, and differentiation due to its role in protein tagging and degradation 3. Dysregulation of the ubiquitination system has been implicated as a crucial factor in tumor development and metastasis 4. Within the ubiquitination system, E3 ubiquitin ligases play a critical role 5. Tripartite motif-containing 50 (TRIM50), a member of the E3 ubiquitin ligase family, has garnered considerable attention 6. Multiple studies have demonstrated the significant role of TRIM50 in tumorigenesis and tumor progression. In ovarian cancer, TRIM50 has been found to act as a negative regulator of Src protein, promoting ubiquitin-dependent K48-linked ubiquitination and the subsequent degradation of Src, thereby inhibiting ovarian cancer development 7. Similarly, in pancreatic cancer, TRIM50 promotes the reversal of epithelial-mesenchymal transition (EMT) by degrading Snail1 protein, and its decreased expression level correlates with poor prognosis 8. A recent study has also identified the inhibitory role of TRIM50 in the progression of gastric cancer 9. These findings highlight TRIM50 as an important TSG that exerts crucial negative regulatory effects on tumor development and metastasis.

Increasing amounts of evidence support the idea that cancer is a metabolic disease and that cancer progression relies on metabolic reprogramming, which is considered a hallmark of cancer 10, 11. Aerobic glycolysis is the primary pathway by which tumor cells utilize glucose, known as the Warburg effect 12, 13. Numerous studies have indicated that tumor cells modulate the expression of glycolysis-related proteins through ubiquitin modification, thereby activating or inhibiting the glycolytic pathway 14, 15, 16. For instance, the E3 ligase Fbw7 inhibits glycolysis in diffuse large B-cell lymphoma by promoting the ubiquitin-mediated degradation of LDHA 14. HectH9, through K63-linked ubiquitination, facilitates the transfer of HK2 to mitochondria and enhances glycolysis in prostate cancer 15. Moreover, TRIM21, by means of ubiquitinating hnRNPA1 and thereby inducing degradation, curtails aerobic glycolysis in gastric cancer cells 16. However, further investigation is needed to determine whether TRIM50 can modulate glycolysis in GC cells through its function as a ubiquitin ligase.

In this study, we identified the core E3 ubiquitin ligase TRIM50 in GC tissues. Through rigorous examination and validation using both *in vitro* and *in vivo* models at the cellular, tissue, and animal levels, we elucidated the role of TRIM50 in the progression of GC. Furthermore, for the first time, we elucidated the potential mechanisms by which TRIM50 influences aerobic glycolysis and contributes to GC progression. In conclusion, our findings highlight TRIM50 as a promising and novel target for anticancer therapy.

## Materials and methods

### Patients and tissue samples

A total of 24 pairs of GC patient tumor tissues and adjacent nontumor tissues were collected from the Department of Gastrointestinal Surgery, The First Affiliated Hospital of Nanjing Medical University. Additionally, 124 pairs of GC patient tumor tissues and adjacent nontumor tissues were collected from the Department of Gastrointestinal Surgery, The Affiliated Suzhou Hospital of Nanjing Medical University. These samples were collected between January 2015 and January 2019. Transcriptome microarray analysis was performed on the 24 pairs of tissues from the First Affiliated Hospital using a human gene transcriptome microarray. The sample information can be found in Supplementary Table S1. The 124 pairs of tissues from the Affiliated Suzhou Hospital were mainly used for quantitative real-time polymerase chain reaction (qRT‒PCR) validation and clinical correlation analysis. Clinical and pathological characteristics of the patients, including age, sex, tumor size, T stage, lymph node status, distant metastasis, and TNM stage (AJCC classification), are documented in Supplementary Table S2.

This study was approved by the Ethics Committees of Nanjing Medical University First Affiliated Hospital and Nanjing Medical University Affiliated Suzhou Hospital. Written informed consent was obtained from all patients included in the study.

### Cell culture

The HEK-293T cell line, THP-1 cell line, GES-1 human gastric epithelial cell line, and HGC-27, MKN-45, and AGS human GC cell lines were obtained from the Cell Center at Shanghai Institute of Life Sciences. GES-1, HGC-27, MKN-45, and THP-1 cell lines were cultured in RPMI 1640 medium (without HEPES). AGS cells were cultured in an F-12K nutrient mixture. All culture media were supplemented with 1% penicillin‒streptomycin and 10% fetal bovine serum (FBS). Cells were cultured at 37 °C in a humidified 5% CO2 incubator. The information of cell lines used in this study is listed in Supplementary Table S3.

### RNA extraction and qRT‒PCR

Total RNA extraction from tissues was performed using TRIzol reagent in accordance with the manufacturer's instructions (Invitrogen). TRI Reagent (Ambion) was utilized for isolating total RNA from cultured cell lines. Subsequently, the RNA (500 ng) was reverse-transcribed using reverse transcriptase (Takara). qRT‒PCR analysis was conducted using a Light Cycler (Roche) and a SYBR RT‒PCR kit (Roche). The fold changes in relative transcript abundance were calculated using the 2^(-ΔΔCT)^ method, with GAPDH serving as the internal standard. The sequences of PCR primers are listed in Supplementary Table S4. Data are expressed as the average of at least three independent experimental replicates.

### Protein extraction and Western blotting

Total protein lysates were prepared using a protein extraction kit (KGP9100, Key Gene). Proteins were separated on a 10% SDS‒PAGE gel and transferred onto a polyvinylidene difluoride (PVDF) membrane. After blocking in 5% skim milk in TBST buffer, the membrane was incubated overnight at 4 °C with specific primary antibodies, followed by washing and incubation with secondary antibodies. The signal was visualized using a chemiluminescent HRP substrate (WBKL0100, Millipore) and a chemiluminescence detection system. A Dynabeads Protein G immunoprecipitation kit (Invitrogen) was used to immunoprecipitate cell lysates for immunoprecipitation according to the manufacturer's instructions. Subsequently, the immunoprecipitated proteins were detected by Western blotting. The antibodies used in this study are listed in Supplementary Table S5.

### Statistical analysis

The statistical analysis in this study was performed using GraphPad Prism (version 8.0) and SPSS software (version 23.0). The Shapiro-Wilk test was used for normality testing of quantitative data. Quantitative data with normal distribution were presented as mean ± standard deviation (SD) or mean ± standard error of the mean (SEM). For data that did not meet the criteria for normality, median and interquartile range (IQR) were used to represent the central tendency and dispersion, respectively. Differences between experimental and control groups were analyzed with a two-tailed unpaired Student's t test for parametric tests and Wilcoxon signed-rank test for nonparametric tests. For comparisons among multiple groups, a one-way or two-way ANOVA test was conducted. Spearman's correlation test was utilized to assess the association between TRIM50 expression and clinical pathological features. Kaplan‒Meier analysis was applied to calculate survival rates, and the log-rank test was used to examine differences in survival rates between the two groups. All statistical tests were two-tailed exact tests, with a significance level of p < 0.05.

The additional methods utilized in this study are presented in the supplementary materials.

## Results

### TRIM50 expression is downregulated in GC, and its expression is positively correlated with patient prognosis

**Figure 1:**
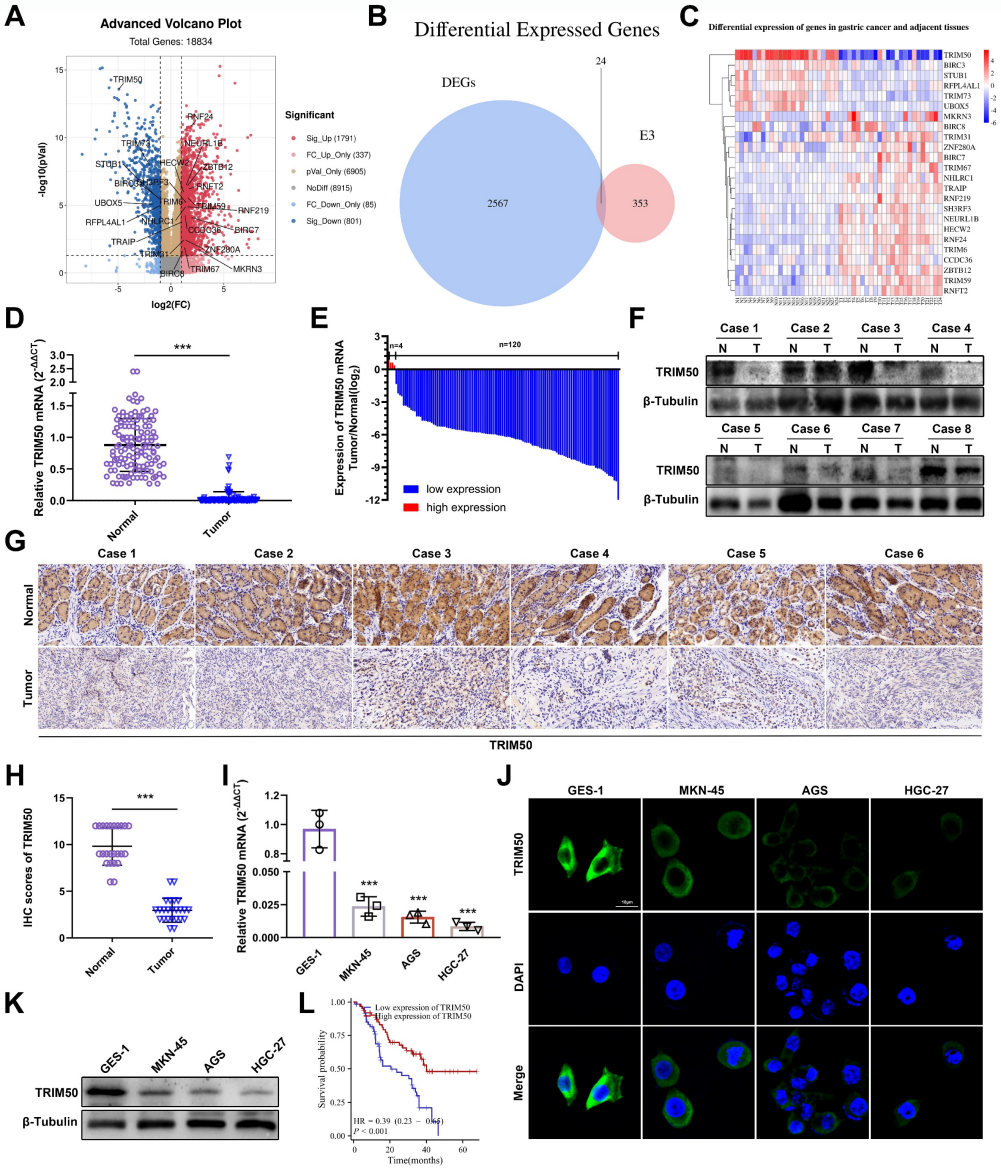
**Expression patterns and clinical significance of TRIM50 in GC. (A)** Volcano plot illustrated the differential expression of mRNA in 24 GC tissues compared to adjacent normal tissues (P < 0.05, Log2FC > 1 or < -1, excluding duplicate values). **(B)** Venn diagram depicted the intersection between differentially expressed genes and the E3 ubiquitin ligase gene family. **(C)** Heatmap presented the mRNA expression levels of the 24 intersected genes, with TRIM50 showing the most pronounced differential expression among them. **(D)** qRT‒PCR demonstrated the variation in TRIM50 mRNA expression between 124 pairs of GC tissues and adjacent non-neoplastic tissues. **(E)** Fold changes (log2) of TRIM50 in each paired sample, arranged in descending order. **(F)** Western blotting revealed the differential expression of the TRIM50 protein in GC tissues and adjacent non-neoplastic tissues. **(G)**,** (H)** Immunohistochemistry and scoring displayed the differential expression of TRIM50 protein in GC tissues and adjacent non-neoplastic tissues (n = 24). Scale bar: 100 μm. **(I)** qRT‒PCR exhibited the expression levels of TRIM50 mRNA in gastric mucosal epithelial cell lines (GES-1) and GC cell lines (MKN-45, AGS, and HGC-27). **(J)** Immunofluorescence demonstrated the protein expression levels of TRIM50 in gastric mucosal epithelial cell lines (GES-1) and GC cell lines (MKN-45, AGS, and HGC-27), with cell nuclei stained using DAPI. Scale bar: 10 μm.** (K)** Western blotting showed the protein expression levels of TRIM50 in gastric mucosal epithelial cell lines (GES-1) and GC cell lines (MKN-45, AGS, and HGC-27). **(L)** Survival analysis of GC patients based on TRIM50 mRNA expression (n = 124). The median expression level of TRIM50 was used as the cut-off value to classify patients into high and low expression groups. The data are representative of three independent experiments. Quantitative data are shown as mean ± SD **(D**, **H**, **I)**, or mean ± SEM **(L)**, and p values were determined by two-tailed unpaired Student's t test **(I**,** D**, **H)**, or log rank test **(L)** (*p < 0.05, **p < 0.01, ***p < 0.001).

To identify the core E3 ubiquitin ligases associated with GC progression, mRNA differential expression analysis was performed on 24 pairs of GC tissues and adjacent noncancerous tissues. A total of 2567 differentially expressed genes were identified (Figure 1A), and among the gene family of E3 ubiquitin ligases, 24 differentially expressed genes were found (Figure 1B), with TRIM50 exhibiting the most significant difference (Figure 1C). qRT‒PCR, Western blotting, and immunohistochemical analyses confirmed lower TRIM50 mRNA and protein expression in GC tissues than in adjacent noncancerous tissues (Figure 1D-H). Similarly, lower levels of TRIM50 expression were observed in GC cell lines than in the gastric epithelial cell line (Figure 1I-K). Clinical pathological parameters showed a negative correlation between TRIM50 expression and tumor volume, distant metastasis, and TNM staging (Table 1, Figure S1A-C). Furthermore, low expression of TRIM50 was associated with a poor prognosis in GC patients (Figure 1L, S1D). These findings suggest that TRIM50 is a potential prognostic biomarker in GC.

### TRIM50 directly inhibits the proliferation of GC cells and indirectly inhibits the invasion and migration of GC cells by suppressing tumor-associated macrophage M2 polarization

**Figure 2:**
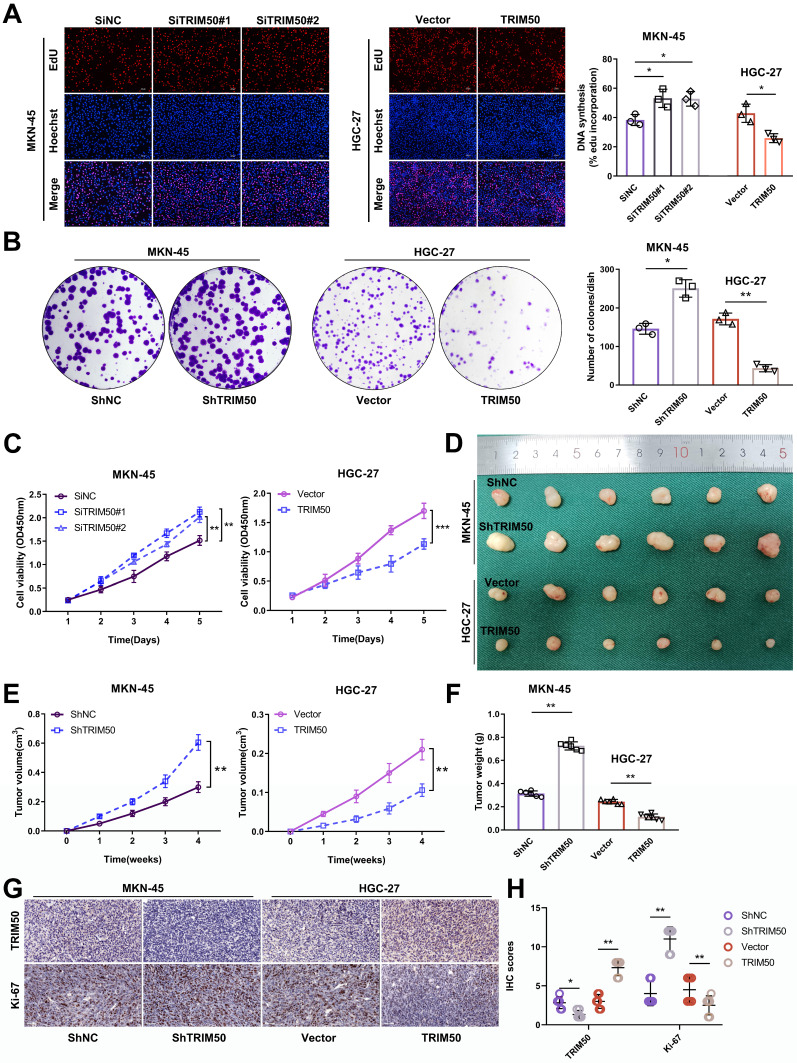
** Suppression of GC cell proliferation by TRIM50. (A)** EdU assay examined the impact of TRIM50 knockdown and overexpression on the proliferation of MKN-45 and HGC-27 cells. Scale bar: 100 μm. **(B)** Colony formation assay investigated the effect of TRIM50 on the proliferation of MKN-45 and HGC-27 cells. **(C)** CCK-8 assay evaluated the influence of TRIM50 on the proliferation of MKN-45 and HGC-27 cells. **(D)** Mouse xenograft model was used to evaluate the effect of TRIM50 on cell proliferation *in vivo*; images of subcutaneous xenograft tumors from mouse models were displayed (n = 6). **(E)** Time-volume curve of subcutaneous xenograft tumors. **(F)** Weight of subcutaneous xenograft tumors. **(G)** Immunohistochemical staining of TRIM50 and Ki-67 in subcutaneous xenograft tumors. **(H)** Immunohistochemical staining score of TRIM50 and Ki-67 in subcutaneous xenograft tumors. The data are representative of three independent experiments. Quantitative data are shown as mean ± SD (**A**, **B**, **F**, **H**), or mean ± SEM (**C**, **E**). p values were determined by two-way ANOVA test (**C**, **E**), or two-tailed unpaired Student's t test (**A**, **B**, **F**, **H**) (*p < 0.05, **p < 0.01, ***p < 0.001).

The differential expression of TRIM50 in the gastric cancer cell lines MKN-45 and HGC-27 facilitated our exploration of the biological functions of TRIM50. This was achieved by downregulating TRIM50 expression in MKN-45 and upregulating it in HGC-27 using siRNAs and overexpression plasmids targeting TRIM50 mRNA and protein expression (Supplementary Tables S5 and S6). The transfection efficiency was validated by qRT‒PCR or Western blotting (Supplementary Figure S2A-D). Subsequent EdU assays, colony formation assays and CCK-8 assays revealed that knockdown of TRIM50 expression promoted MKN-45 cell proliferation, while overexpression of TRIM50 suppressed HGC-27 cell proliferation (Figure 2A-C). *In vivo* experiments confirmed the inhibitory effect of TRIM50 on GC tumor growth (Figure 2D-F), supported by IHC staining showing a negative correlation between TRIM50 and Ki-67 expression (Figure 2G, H).

**Figure 3:**
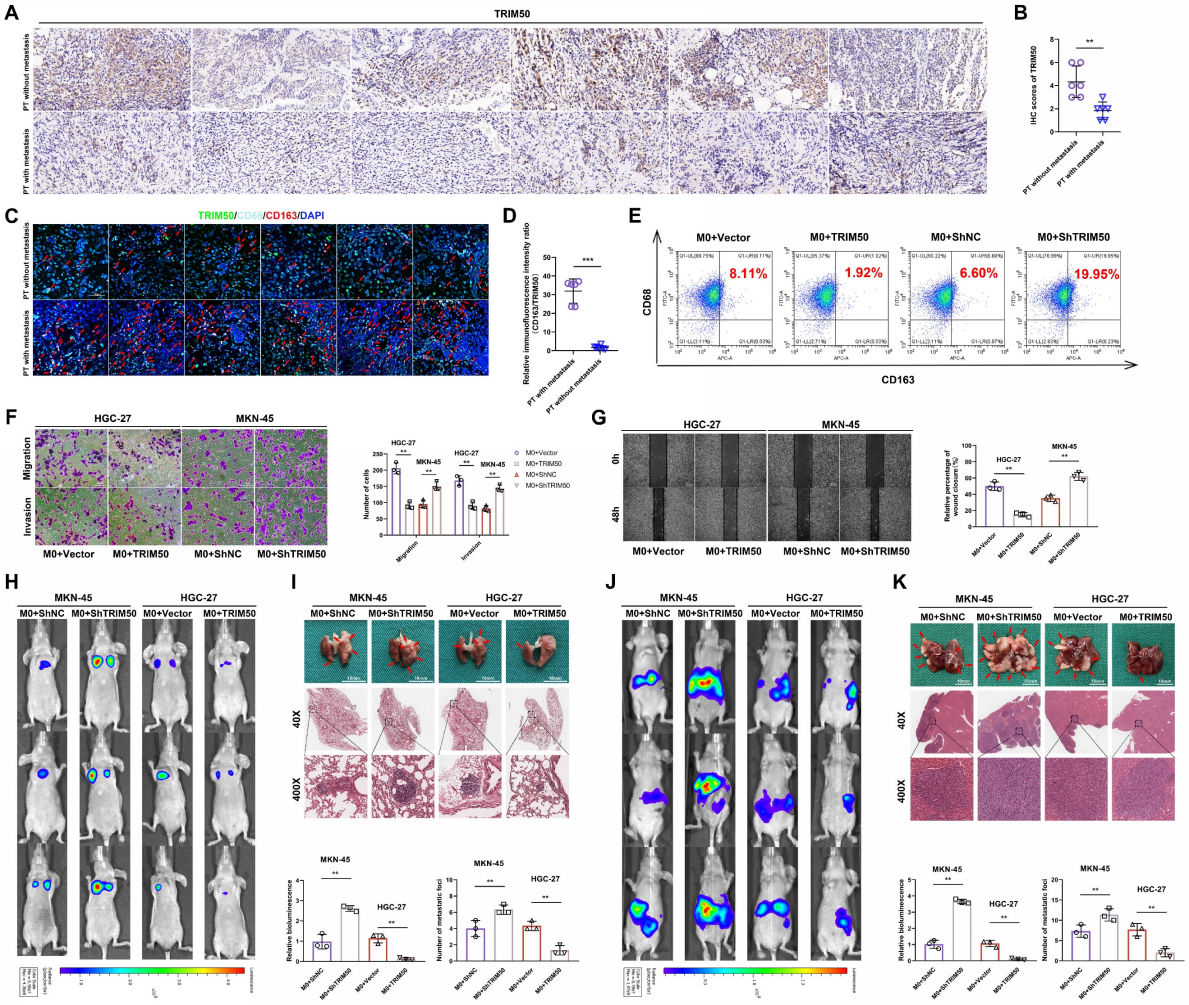
** TRIM50 inhibits the invasion and migration of GC cells by suppressing M2 polarization of tumor-associated macrophages. (A)**,** (B)** Immunohistochemical staining and their scoring showed the expression of TRIM50 in primary tumors of GC patients with and without metastasis. Scale bar: 100 μm. **(C)** Immunofluorescence staining of TRIM50, CD68 (human macrophage marker), and CD163 (M2 marker) in GC tissues with and without distant metastasis, with cell nuclei stained using DAPI. Scale bar: 100 μm. **(D)** Relative fluorescence intensity ratio of CD163/TRIM50 in the tissues. **(E)** Coculture of HGC-27 cells transfected with TRIM50 overexpression vectors or MKN-45 cells transfected with TRIM50 knockdown vectors with M0 macrophages, followed by flow cytometry analysis of CD68 (human macrophage marker) and CD163 (M2 marker) expression on macrophages. **(F)**,** (G)** Collection of macrophage supernatant from each well and addition to the culture medium of human GC cells (HGC-27, MKN-45). Transwell (F) and wound healing assays (G) examined changes in the migration and invasion ability of GC cells. Scale bar: 100 μm (F), 200 μm (G). **(H)** Mouse lung metastasis model was used to evaluate the indirect effect of TRIM50 on cell metastasis *in vivo*. IVIS was used to detect the values of bioluminescence imaging signals in lung metastases. Relative bioluminescence was expressed as mean ± standard deviation (n = 3). **(I)** Lung metastasis and H&E staining. **(J)** Mouse liver metastasis model was used to evaluate the indirect effect of TRIM50 on cell metastasis *in vivo*. IVIS was used to detect the values of bioluminescence imaging signals in liver metastases. Relative bioluminescence was expressed as mean ± standard deviation (n = 3). **(K)** Liver metastasis and HE staining. The data are representative of three independent experiments. Quantitative data are shown as mean ± SD (**B**, **D**, **F**, **G**, **H**, **I**, **J**, **K**). p values were determined by two-tailed unpaired Student's t test (*p < 0.05, **p < 0.01, ***p < 0.001).

Transwell and wound healing assays were utilized to evaluate the impact of TRIM50 on the migratory and invasive capabilities of GC cells, with negligible effects observed (Figure S3A, B). Lower TRIM50 expression was observed in the primary tumors of GC patients with distant metastasis than in those without metastasis, suggesting that TRIM50 may inhibit GC metastasis through indirect mechanisms (Figure 3A, B). Given our earlier research that GC cells can promote metastasis by inducing M2 polarization in macrophages 17, we investigated whether TRIM50 could mediate this polarization process. Through tissue immunofluorescence, we examined the relationship between TRIM50 expression and M2 macrophage infiltration in GC. Notably, we observed lower TRIM50 expression and increased M2 macrophage infiltration in primary tumors of GC patients with metastasis compared to those without metastasis (Figure 3C, D), highlighting a potential role for TRIM50 in the modulation of the tumor immune microenvironment and the GC progression.

To elucidate the underlying mechanism, a coculture model of GC cells and M0 macrophages was established (Figure S4). Coculturing TRIM50-overexpressing HGC-27 cells with M0 macrophages decreased M2 macrophage polarization, while coculturing TRIM50-knockdown MKN-45 cells with M0 macrophages increased M2 macrophage polarization (Figure 3E). Supernatants from the macrophage cultures were collected and added to the culture medium of GC cells. The supernatant from the coculture of TRIM50-overexpressing HGC-27 cells and macrophages reduced GC cell invasion and migration, while the supernatant from the coculture of TRIM50-knockdown MKN-45 cells and macrophages had the opposite effects (Figure 3F, G). *In vivo* experiments confirmed that TRIM50 can indirectly inhibit GC metastasis to the lung and liver by modulating macrophage polarization (Figure 3H-K).

### TRIM50 suppresses glycolysis to directly inhibit GC cell proliferation and indirectly inhibit their invasion and migration

**Figure 4:**
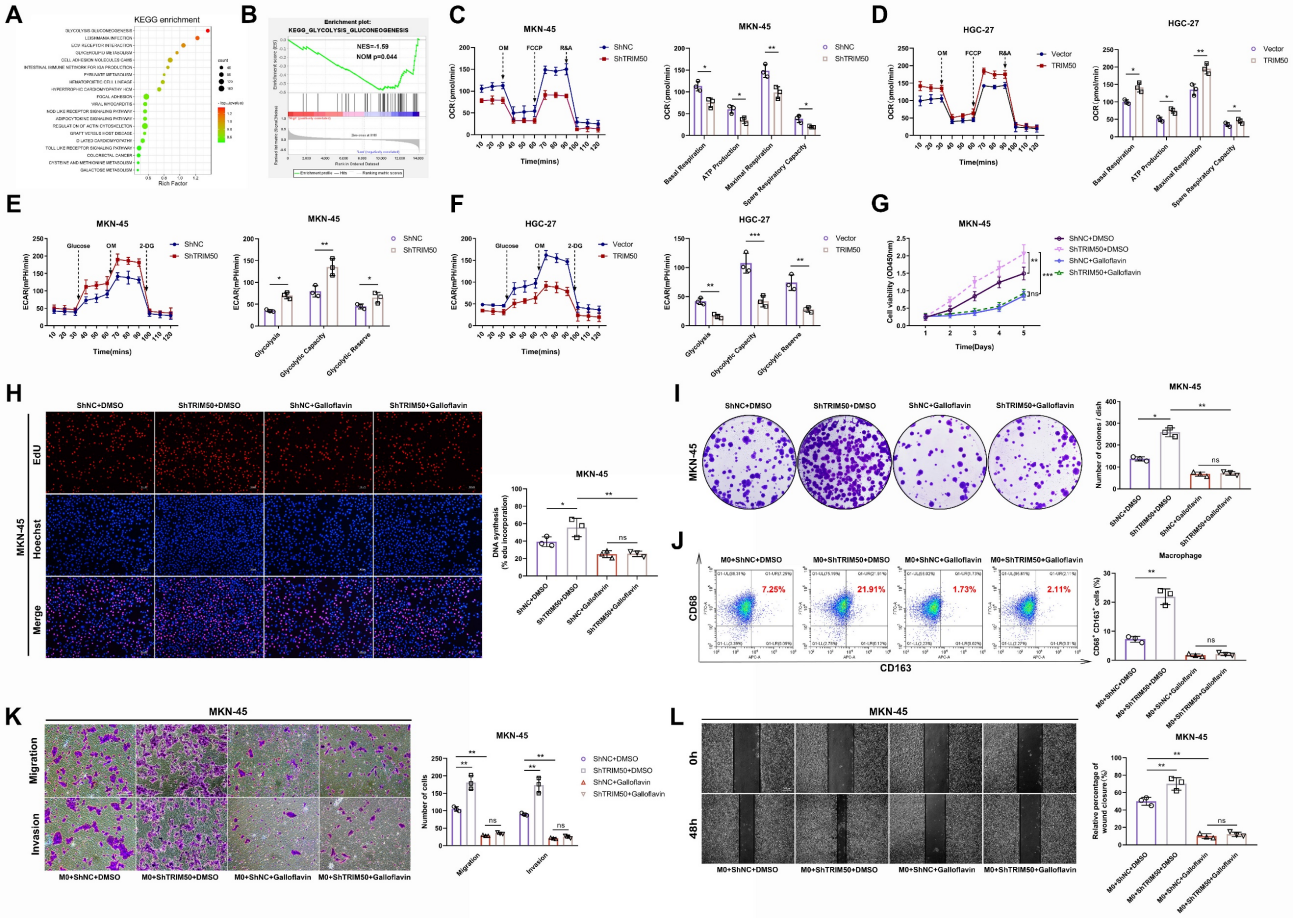
** TRIM50 suppresses glycolysis to directly inhibit GC cell proliferation and indirectly inhibit their invasion and migration. (A)**,** (B)** Gene Set Enrichment Analysis (GSEA) based on our mRNA array results. Specifically, TRIM50 expression in the mRNA array served as the phenotype label, and all mRNA data were considered as gene sets. **(C)**,** (D)** Assessment of TRIM50's impact on oxygen consumption rate using the Seahorse Energy Flux system by measuring OCR, OM (oligomycin), FCCP (Carbonyl cyanide 4-(trifluoromethoxy)phenylhydrazone), and R&A (Rotenone & Antimycin A). **(E)**,** (F)** Assessment of TRIM50's impact on glycolysis rate using the Seahorse Energy Flux system by measuring ECAR, OM (oligomycin), and 2-DG (2-deoxyglucose). **(G)** CCK-8 assay evaluated the effect of TRIM50 knockdown and Galloflavin (a glycolysis inhibitor at 50 μM) on the proliferation of MKN-45 cells. **(H)** EdU assay assessed the effect of TRIM50 knockdown and Galloflavin on the proliferation of MKN-45 cells. Scale bar: 50 μm. **(I)** Colony formation assay examined the effect of TRIM50 knockdown and Galloflavin on the proliferation of GC cells (MKN-45). **(J)** Coculture of intervened MKN-45 cells with M0 macrophages, followed by flow cytometry analysis of CD68 (human macrophage marker) and CD163 (M2 marker) expression on macrophages. **(K)**,** (L)** Addition of intervened macrophage supernatant to the culture medium of GC cells (MKN-45), followed by Transwell (K) and wound healing assay (L) to assess the invasion and migration ability of GC cells (MKN-45). Scale bars: 100 μm (K), 200 μm (L). The data are representative of three independent experiments. p values were determined by two-way ANOVA test (**G**), or two-tailed unpaired Student's t test (**C**, **D**, **E**, **F**, **H**, **I**, **J**, **K**, **L**) (*p < 0.05, **p < 0.01, ***p < 0.001).

To explore the downstream regulatory mechanisms affected by TRIM50, we conducted gene set enrichment analysis and found a negative correlation between TRIM50 expression and the regulation of the glycolytic pathway (p = 0.044, Figure 4A, B). OCR and ECAR experiments confirmed that TRIM50 enhances mitochondrial respiration (Figure 4C, D) and inhibits glycolysis in GC cells (Figure 4E, F). We treated GC cells with the glycolysis inhibitor galloflavin (50 μM) and observed that it suppressed the increase in proliferation caused by TRIM50 knockdown, whereas the knockdown of TRIM50 did not induce any changes in cell proliferation under the precondition of glycolysis inhibition (Figure 4G-I), indicating that TRIM50 inhibits cell proliferation through the suppression of glycolysis. Additionally, the glycolysis inhibitor inhibited M2 macrophage polarization induced by TRIM50 knockdown and suppressed the invasive and migratory abilities of GC cells in the presence of macrophage supernatant (Figure 4K, L). However, the knockdown of TRIM50 in GC cells did not significantly affect M2 macrophage polarization (Figure 4J) or the invasive and migratory abilities of GC cells under the precondition of glycolysis inhibition (Figure 4K, L). Thus, it can be inferred that TRIM50 directly inhibits GC cell proliferation and indirectly inhibits invasion and migration by suppressing glycolysis within GC cells.

**Figure 5:**
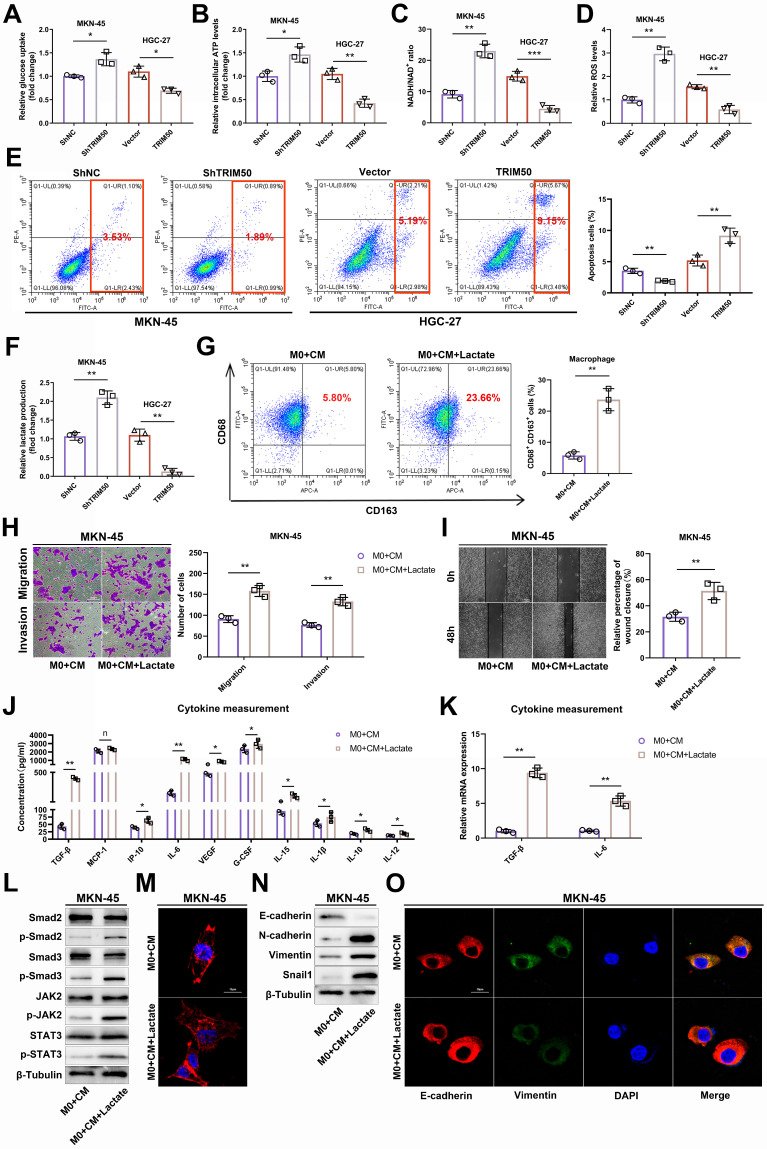
** The mechanism by which TRIM50 directly inhibits GC cell proliferation and indirectly inhibits invasion and migration through the glycolytic pathway. (A)** Impact of TRIM50 knockdown and overexpression on glucose uptake in MKN-45 and HGC-27 cells. **(B)** Effect of TRIM50 modulation on ATP production in MKN-45 and HGC-27 cells. **(C)** The influence of TRIM50 on NADH/NAD+ ratios in MKN-45 and HGC-27 cells. **(D)** Effect of TRIM50 on ROS accumulation in MKN-45 and HGC-27 cells. **(E)** The role of TRIM50 in regulating apoptosis in MKN-45 and HGC-27 cells. **(F)** Lactate production in response to TRIM50 modulation in MKN-45 and HGC-27 cells. **(G)** M2 macrophage polarization in THP-1 cells induced with PMA and treated with lactate, analyzed by flow cytometry. **(H)**,** (I)** The migration and invasive capabilities of MKN-45 cells treated with lactate-conditioned macrophage supernatant, assessed by Transwell (H) and wound healing (I) assays. Scale bars: 100 μm (H), 200 μm (I). **(J)** ELISA assay measured the concentration of tumor invasion and migration-related cytokines (TGF-β, MCP-1, IP-10, IL-6, VEGF, G-CSF, IL-15, IL-1β, IL-10, and IL-12) in lactate-trained macrophage conditioned medium, with TGF-β and IL-6 showing marked changes. **(K)** qRT‒PCR analysis of TGF-β and IL-6 mRNA in lactate-conditioned macrophage media. **(L)** Western blotting analysis of TGF-β and JAK-STAT pathway protein phosphorylation in MKN-45 cells treated with lactate-conditioned supernatant. **(M)** Morphological changes of GC cells cultured with lactate-trained macrophage supernatant observed by immunofluorescence staining of the cytoskeleton using phalloidin, with DAPI staining for cell nuclei. Scale bar: 20 μm. **(N)**,** (O)** Analysis of EMT marker protein expression in MKN-45 cells with lactate-conditioned macrophage supernatant by Western blotting (N) and immunofluorescence staining (O), with DAPI staining for cell nuclei. Scale bar: 20 μm. The data are representative of three independent experiments. p values were determined by two-tailed unpaired Student's t test (*p < 0.05, **p < 0.01, ***p < 0.001).

To further elucidate the underlying mechanisms, we initially observed that TRIM50 effectively inhibits glucose uptake and ATP generation in GC cells (Figure 5A, B), thereby reducing the amount of energy synthesis needed for tumor cell proliferation. Moreover, changes in TRIM50 expression affected the NADH/NAD+ ratio (Figure 5C) and cellular ROS levels (Figure 5D), regulating apoptosis levels through oxidative stress responses (Figure 5E), further contributing to the inhibition of GC cell proliferation. Lactate, as the end product of glycolysis, plays a vital role in immune cell activation and differentiation in the tumor microenvironment.

We found that TRIM50 inhibited lactate production by GC cells (Figure 5F). Furthermore, we demonstrated that lactate promotes M2 macrophage polarization (Figure 5G), and the addition of supernatant from lactate-trained macrophages significantly enhanced the invasive and migratory abilities of MKN-45 cells (Figure 5H, I). Analysis of cytokine concentrations revealed elevated levels of TGF-β, IP-10, IL-6, VEGF, G-CSF, IL-15, IL-1β, IL-10, and IL-12 in the supernatant from lactate-trained macrophage cultures, with TGF-β and IL-6 displaying the most prominent alterations in expression (Figure 5J, K). Considering that TGF-β and IL-6 can activate the TGF-β and JAK/STAT signaling pathways, thereby promoting the EMT of GC cells, we performed Western blotting to examine the phosphorylation status of key proteins in these signaling pathways. We observed a notable upregulation of phosphorylated Smad2/Smad3 and JAK2/STAT3 in GC cells cultured with the supernatant of lactate-trained macrophages (Figure 5L), indicating that the supernatant could activate the TGF-β and JAK/STAT signaling pathways. Culturing GC cells in the supernatant of lactate-trained macrophages led to an EMT morphological transition (Figure 5M) and changes in the protein levels of EMT markers in GC cells (Figure 5N, O).

### TRIM50 inhibits glycolysis and malignant biological behavior in GC cells by downregulating PGK1 expression

**Figure 6:**
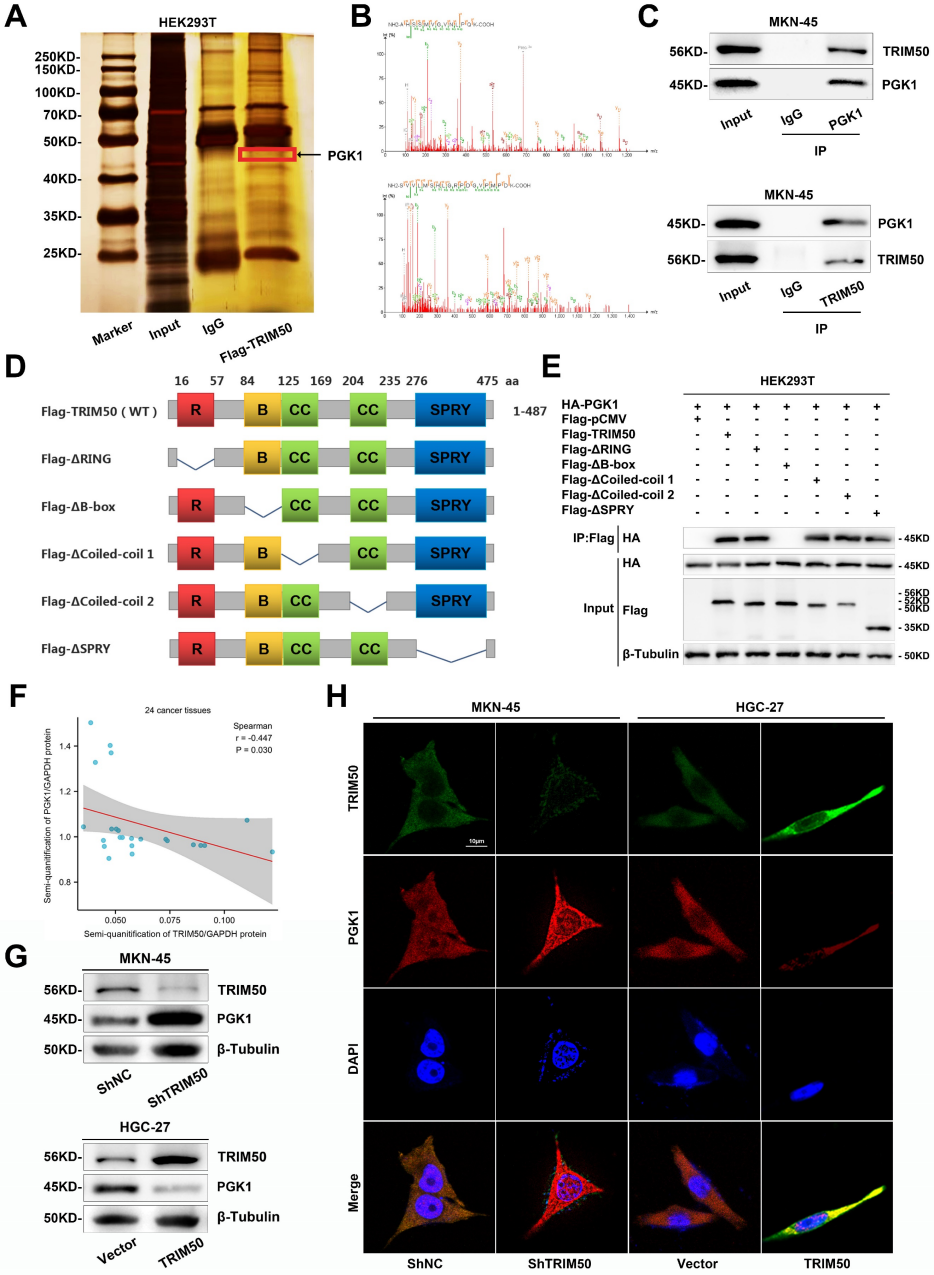
** Interaction between TRIM50 and PGK1 and regulation of PGK1 expression. (A)** Coimmunoprecipitation (Co-IP) of TRIM50-FLAG plasmid transfected into GC cells. Silver staining analysis of the pulled-down proteins. The image showed the silver-stained gel, with the target protein location marked by red boxes.** (B)** Secondary structure of PGK1 pulled down by TRIM50 in mass spectrometry. **(C)** Confirmation of the interaction between TRIM50 and PGK1 through reciprocal Co-IP and Western blotting. **(D)** Schematic representation of the TRIM50 structure domains and its structural domain-deleted mutants (Flag-ΔRING, Flag-ΔB-box, Flag-ΔCoiled-coil 1, Flag-ΔCoiled-coil 2, Flag-ΔSPRY) plasmid constructs. **(E)** HEK293T cells were transfected with HA-PGK1 plasmid together with wild-type Flag-TRIM50 plasmid or its structural domain-deleted mutants. The interaction between HA-PGK1 and wild-type Flag-TRIM50 or its mutants was detected by immunoprecipitation. **(F)** Protein semi-quantitative analysis of 24 tumor tissues revealed the correlation between PGK1 protein expression and TRIM50 protein expression, calculated by Spearman correlation analysis with statistical significance determined using a two-tailed unpaired Student's t-test (*p < 0.05, **p < 0.01, ***p < 0.001). **(G)** Western blotting confirmed that TRIM50 knockdown increased the protein level of PGK1 in MKN-45 cells, while TRIM50 overexpression decreased the protein level of PGK1 in HGC-27 cells. **(H)** Immunofluorescence observation of the effect of TRIM50 knockdown and overexpression on the protein level of PGK1 in GC cells, with cell nuclei stained with DAPI. Scale bar: 10 μm.

To further elucidate the specific mechanism by which TRIM50 inhibits the glycolytic pathway in GC cells, we conducted Co-IP and mass spectrometry analysis, thereby revealing the potential interaction between TRIM50 and PGK1 (Figure 6A-B). Notably, PGK1 is a key enzyme in glycolysis and emerged as the top-ranked glycolytic enzyme in the Co-IP and mass spectrometry analysis of TRIM50. Furthermore, high expression of PGK1 was associated with a poor prognosis in GC patients (Figure S5A, B). Consequently, we have identified PGK1 as the prime candidate for our subsequent study. Reciprocal Co-IP experiments further confirmed their binding interaction (Figure 6C). We generated TRIM50 domain deletion mutants (Figure 6D) and found that the B-box domain of TRIM50 is essential for its interaction with PGK1 (Figure 6E). Subsequently, protein semiquantification analysis of 24 tumor tissues demonstrated a negative correlation between the expression of PGK1 protein and TRIM50 protein (Figure 6F). Western blotting and immunofluorescence assays revealed that overexpression of TRIM50 led to downregulation of PGK1 protein (Figure 6G, H).

**Figure 7:**
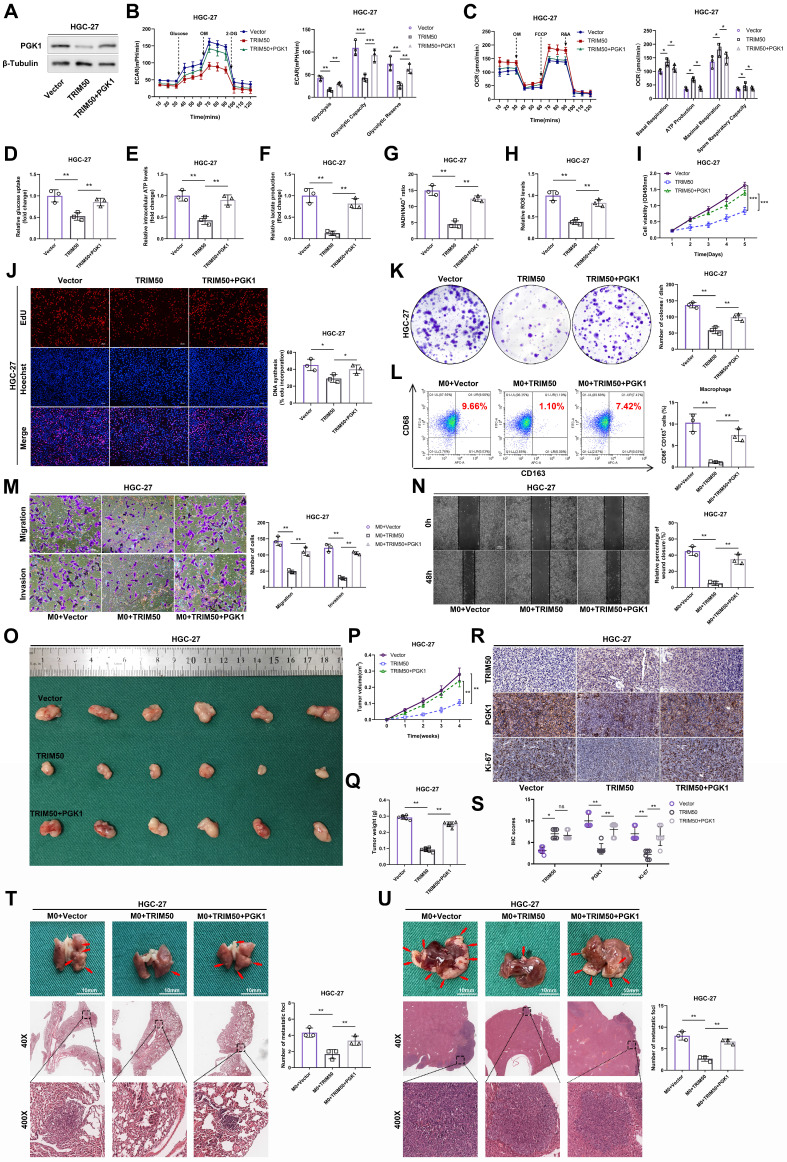
** Rescue of glycolysis and cellular malignancy in TRIM50-overexpressing GC cells by PGK1 restoration. (A)** Western blotting analysis of PGK1 expression in HGC-27-TRIM50 cells. **(B)** ECAR assay showed that PGK1 restoration reversed the inhibitory effect of TRIM50 on glycolytic capacity in HGC-27 cells overexpressing TRIM50. **(C)** OCR assay showed that PGK1 restoration reversed the promoting effect of TRIM50 on mitochondrial oxidative phosphorylation capacity in HGC-27 cells overexpressing TRIM50. **(D)**-**(H)** Restoration of PGK1 expression reversed the inhibitory effects of TRIM50 overexpression on glucose uptake (D), ATP levels (E), lactate production (F), NADH/NAD+ conversion (G), and ROS levels (H) in HGC-27 cells overexpressing TRIM50. **(I)** CCK-8 assay, **(J)** EdU assay and **(K)** colony formation assay demonstrated that restoration of PGK1 expression reverses the inhibitory effect of TRIM50 on the proliferation capacity of HGC-27 cells. Scale bar: 100 μm (J). **(L)** Flow cytometry analysis showed that restoration of PGK1 expression reverses the M2 polarization of macrophages cocultured with HGC-27 cells overexpressing TRIM50. **(M)**, **(N)** Transwell (M) and wound healing assay (N) demonstrated that restoration of PGK1 expression reverses the indirect inhibitory effect of TRIM50 on the invasion and migration capacity of GC cells cultured with macrophage-conditioned medium. Scale bar: 100 μm (M), 200 μm (N). **(O)** The mouse model of subcutaneous xenograft tumor was used to evaluate the effect of restoring PGK1 expression on reversing the direct inhibition of tumor growth caused by TRIM50 overexpression (n = 6). **(P)** Time-volume curve of subcutaneous xenograft tumors. **(Q)** Weight of subcutaneous xenograft tumors. **(R)** Immunohistochemical staining of TRIM50, PGK1, and Ki-67 in subcutaneous xenograft tumors was performed, with **(S)** showing the quantified staining scores. **(T)**, **(U)** Mouse models of lung (T) and liver metastasis (U) were used to evaluate how PGK1 restoration reversed the indirect inhibitory effects of TRIM50 on metastasis, with H&E staining for evaluation (n=3 for each).The data are representative of three independent experiments. p values were determined by two-way ANOVA test (**I**, **P**), or two-tailed unpaired Student's t test (**B**, **C**, **D**, **E**, **F**, **G**, **H**, **J**, **K**, **L**, **M**, **N**, Q, **S**, **T**, **U**) (*p < 0.05, **p < 0.01, ***p < 0.001)

To validate PGK1 as a target of TRIM50, we restored PGK1 expression in TRIM50-overexpressing cells (Figure 7A). This restoration reversed the inhibitory effects of TRIM50 on glycolytic capacity, mitochondrial oxidative phosphorylation, glucose uptake, intracellular ATP levels, lactate production, NAD+/NADH conversion, and intracellular ROS levels (Figure 7B-H), ultimately reversing the inhibitory effect of TRIM50 on GC cell proliferation (Figure 7I-K). Similarly, restoring PGK1 also reversed the inhibitory effect of TRIM50 on M2 polarization of macrophages (Figure 7L). Furthermore, adding the supernatant of trained macrophages to the medium of GC cells reversed the indirect inhibitory effects of TRIM50 on cell invasion and migration (Figure 7M, N). The subcutaneous xenograft tumor model and metastasis models confirmed the reversal of the inhibitory effects of TRIM50 on GC growth and metastasis by restoring PGK1 expression (Figure 7O-U). Similarly, in TRIM50-knockdown cells, silencing PGK1 expression attenuated the effects of TRIM50 knockdown on glycolytic capacity, mitochondrial oxidative phosphorylation, glucose uptake, ATP levels, lactate production, NAD+/NADH conversion, intracellular ROS levels, GC cell proliferation, M2 polarization of M0 macrophages, and GC cell invasion and migration (Figure S6A-N). The subcutaneous xenograft tumor model and metastasis models yielded consistent results (Figure S6O-U). These results support PGK1 as a downstream target of TRIM50 in regulating glycolysis, proliferation, and metastasis in GC cells.

### TRIM50 induces PGK1 degradation through ubiquitination

**Figure 8:**
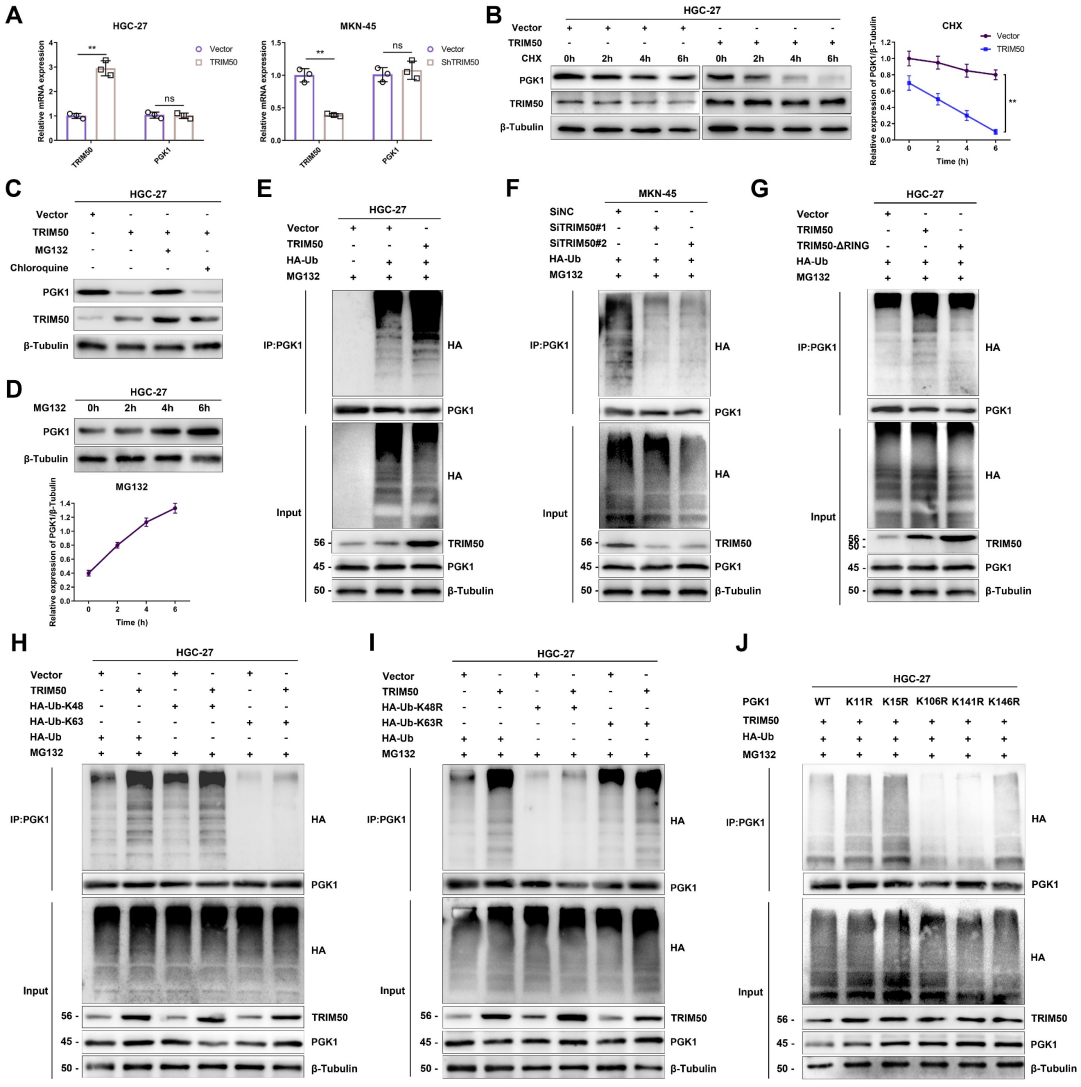
** TRIM50 induces the degradation of PGK1 through ubiquitination modification. (A)** qRT‒PCR analysis demonstrated that PGK1 mRNA levels remained unchanged following TRIM50 overexpression or knockdown, suggesting post-transcriptional regulation. **(B)** In HGC-27 cells, the degradation rate of PGK1 protein was significantly accelerated when TRIM50 was overexpressed, as shown by Western blotting analysis after treatment with cycloheximide (CHX) at various time points. **(C)** The proteasome inhibitor MG132, but not the lysosome inhibitor chloroquine, reduced the degradation rate of PGK1 in TRIM50-overexpressing cells, indicating proteasome-mediated degradation. **(D)** The addition of MG132 led to a time-dependent increase in PGK1 protein levels, further supporting proteasomal degradation. **(E)** Immunoprecipitation (IP) analysis using HA-Ub plasmid in HGC-27 cells with TRIM50 overexpression showed increased ubiquitination of PGK1. **(F)** Immunoprecipitation analysis using HA-Ub plasmid in MKN-45 cells with TRIM50 knockdown demonstrated a decrease in PGK1 ubiquitination, indicating that TRIM50 is required for PGK1 ubiquitination. **(G)** Co-immunoprecipitation of TRIM50 with HA-Ub in HGC-27 cells, using a TRIM50 RING domain deletion mutant, confirmed the necessity of the RING domain for the interaction and the role of TRIM50 in PGK1 ubiquitination. **(H)** Co-transfection of TRIM50 with HA-UB-K48 or HA-UB-K63 in HGC-27 cells, followed by immunoprecipitation analysis, further validated the ubiquitination process. **(I)** Co-transfection with TRIM50 and mutant HA-UB plasmids (K48R or K63R) in HGC-27 cells confirmed the specificity of the ubiquitination process. **(J)** Co-transfection with TRIM50 and PGK1 ubiquitination site mutants in HGC-27 cells, followed by immunoprecipitation, identified the critical lysine residues involved in PGK1 ubiquitination. The data are representative of three independent experiments. Quantitative data are shown as mean ± SEM (**B**, **D**), or mean ± SD (**A**). p values were determined by two-way ANOVA test (**B**), or two-tailed unpaired Student's t test (**A**) (*p < 0.05, **p < 0.01, ***p < 0.001)

We previously observed a negative correlation between the protein expression of PGK1 and TRIM50. However, qRT‒PCR analysis revealed that altering TRIM50 expression did not affect the mRNA levels of PGK1 (Figure 8A), indicating that TRIM50 primarily influences posttranslational modifications of PGK1. Treatment with the protein synthesis inhibitor CHX showed that TRIM50 reduced the protein stability of PGK1, resulting in its degradation (Figure 8B). The addition of a lysosome inhibitor (chloroquine) or proteasome inhibitor (MG132) in HGC-27 cell culture confirmed that TRIM50-mediated degradation of PGK1 occurs through the proteasome system rather than the lysosome (Figure 8C). The protein level of PGK1 increased with increasing duration of MG132 treatment, supporting the evidence of PGK1 degradation via the proteasome system (Figure 8D). Considering TRIM50 to be an E3 ubiquitin ligase, we hypothesize that TRIM50 promotes the ubiquitination of PGK1, leading to its degradation through the proteasome system. Ubiquitination assays confirmed that TRIM50 indeed promotes the ubiquitination of PGK1 (Figure 8E, F).

To further elucidate the ubiquitination pattern mediated by TRIM50, we generated RING domain deletion mutants and transfected them into GC cells. Immunoprecipitation analysis confirmed that the conventional RING domain of TRIM50 plays a primary role in binding to ubiquitin (Figure 8G). To further investigate the ubiquitin-binding sites on PGK1, we constructed overexpression and mutant variants targeting the K48 and K63 ubiquitination sites. After transfecting these vectors into GC cells, immunoprecipitation analysis confirmed that the K48 ubiquitination site is primarily responsible for interacting with PGK1, leading to its recognition by the proteasome and subsequent degradation (Figure 8H, I). Finally, to identify the specific sites of PGK1 ubiquitination, we utilized the PLMD website (http://plmd.biocuckoo.org/) and the PhosphoSite website (https://www.phosphosite.org/) for site prediction. We identified five potential ubiquitin-binding sites on PGK1 that could induce its degradation. Subsequently, we constructed mutant variants targeting these sites and transfected them into GC cells. Through immunoprecipitation analysis, we discovered that the primary sites of PGK1 ubiquitination are K106 and K141 (Figure 8J).

### m6A methylation mediates the downregulation of TRIM50 in GC cells

**Figure 9:**
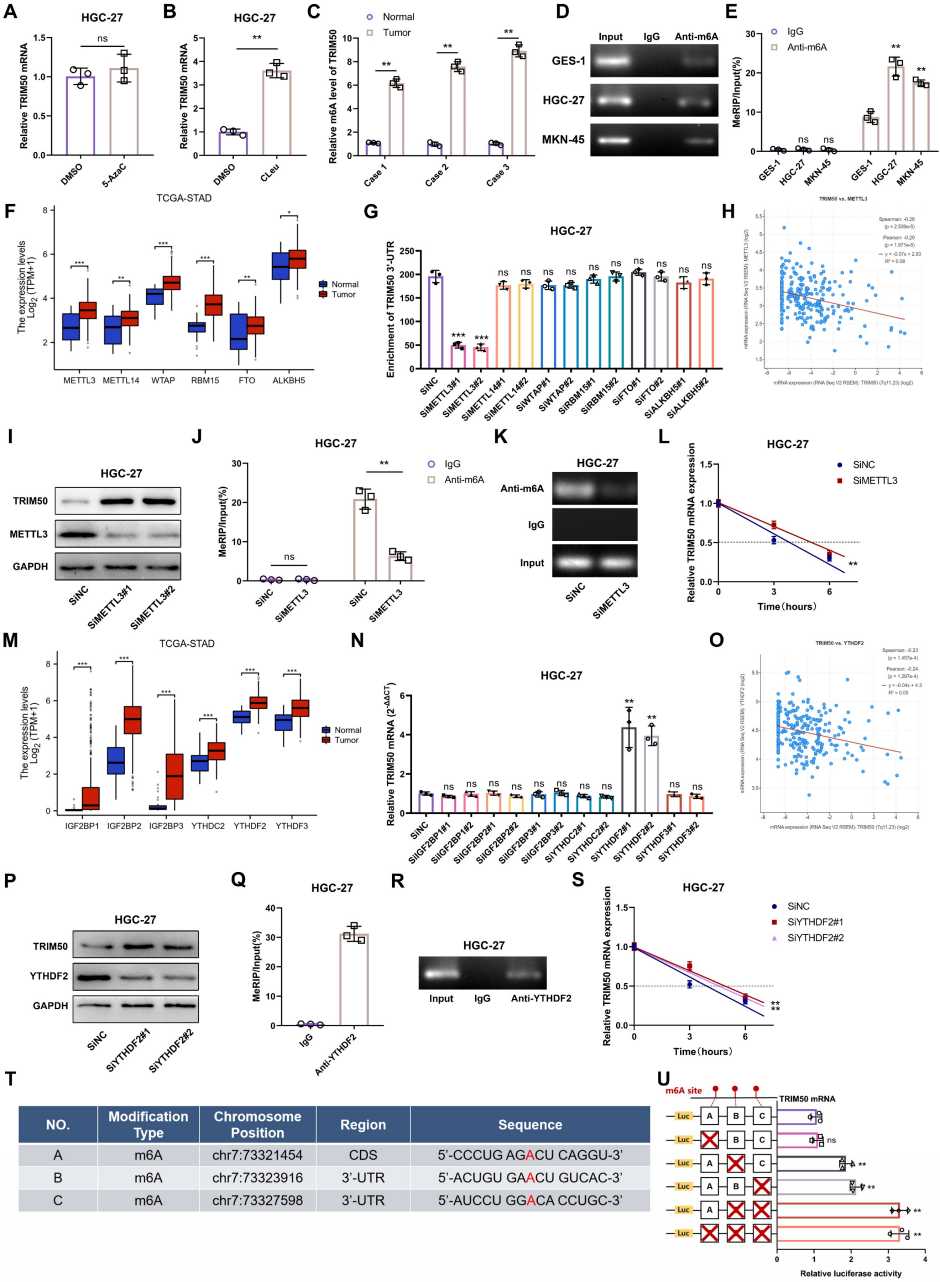
** m6A methylation modulates TRIM50 expression in GC cells. (A)** The qRT‒PCR analysis did not detect any statistically significant changes in the expression of the TRIM50 mRNA following treatment with 5-azacytidine (5-AzaC), an inhibitor of DNA methylation, in the HGC-27 GC cells. **(B)** qRT‒PCR analysis revealed a distinct alteration in the expression of TRIM50 mRNA upon treatment with cycloleucine (CLeu), an inhibitor of the m6A methylation, in HGC-27 GC cells. **(C)** m6A sequencing distinguished m6A patterns between GC and normal tissues. **(D)** Agarose gel electrophoresis analysis confirmed the RNA integrity. **(E)** MeRIP-qRT‒PCR detected variable m6A modification in the TRIM50 3'-UTR among GES-1 and GC cell lines (HGC-27, MKN-45). **(F)** TCGA data analyzed expression levels of m6A writers (methyltransferases) and erasers (demethylases) in GC tissues and adjacent tissues. **(G)** METTL3 knockdown significantly reduced m6A levels in the TRIM50 3'-UTR. **(H)** Spearman correlation identified a link between METTL3 and TRIM50 expression in TCGA's GC data. **(I)-(K)** Western blotting (I), MeRIP (J) and Agarose gel electrophoresis analysis (K) confirmed the impact of METTL3 on TRIM50 protein and mRNA levels. **(L)** Actinomycin D assay assessed mRNA stability post-METTL3 knockdown. **(M)** TCGA data revealed expression levels of m6A reader proteins in GC tissues and adjacent tissues. **(N)** Knockdown of these readers influenced TRIM50 mRNA expression. **(O)** Spearman correlation identified a link between YTHDF2 and TRIM50 expression in TCGA's GC data. **(P)-(R)** Western blotting (P), MeRIP (Q) and Agarose gel electrophoresis analysis (R) confirmed the impact of YTHDF2 on TRIM50 protein and mRNA levels. **(S)** Actinomycin D assay assessed mRNA stability post-YTHDF2 knockdown. **(T)** Prediction of potential m6A modification sites on TRIM50 mRNA was performed using m6A-Atlas databases, and the intersection of these predictions was determined. **(U)** Luciferase reporter gene assays confirmed the functional significance of the primary m6A site. The data are representative of three independent experiments. Quantitative data are shown as mean ± SEM (**L**, **S**), or mean ± SD (**A**, **B**, **C**, **E**, **F**, **G**, **J**, **M**, **N**, **Q**, **U**). p values were determined by two-way ANOVA test (**L**, **S**), two-tailed unpaired Student's t test (**A**, **B**, **C**, **E**, **F**, **G**, **H**, **J**, **M**, **N**, **O**, **Q**, **U**) (*p < 0.05, **p < 0.01, ***p < 0.001)

Aberrant methylation of genes is closely associated with abnormal gene expression. To investigate the underlying cause of TRIM50 underexpression in GC cells, we examined the impact of DNA methylation and RNA methylation. Treatment with the DNA methylation inhibitor 5-azacytidine had no significant effect on TRIM50 mRNA expression, while the m6A methylation inhibitor cycloleucine significantly affected TRIM50 mRNA levels (Figure 9A, B).

This suggests that the m6A methylation levels of TRIM50 are closely associated with its mRNA expression. Sequencing analysis of m6A levels in GC tissues revealed increased m6A motif enrichment in the 3'-UTR of TRIM50 compared to adjacent noncancerous tissues (Figure 9C). This finding was further validated in GES-1, HGC-27, and MKN-45 cell lines (Figure 9D, E), indicating that elevated m6A levels may contribute to the downregulation of TRIM50 in GC.

To further investigate the “writers” and “erasers” involved in the m6A modification of TRIM50 mRNA, we analyzed the TCGA database and found increased expression of the common m6A “writers” METTL3, METTL14, METTL16, RBM15, WATP, and VIRMA and “erasers” FTO and ALKBH5 in GC tissues (Figure 9F). Knockdown of these “writers” and “erasers” in GC cell lines revealed that downregulation of METTL3 expression significantly reduced the m6A levels of TRIM50 mRNA (Figure 9G, S7A). The TCGA analysis also showed a negative correlation between METTL3 expression and TRIM50 in GC tissues (Figure 9H). Decreased METTL3 levels led to increased protein levels of TRIM50 in GC cells (Figure 9I, S7B). MeRIP assays confirmed the role of downregulated METTL3 in reducing m6A modification in TRIM50 mRNA (Figure 9J, 9K, S7C, S7D). Furthermore, the actinomycin D experiment demonstrated that the stability of TRIM50 mRNA was enhanced in GC cells following METTL3 knockdown (Figure 9L, S7E). Moreover, we examined the expression of six stability-related m6A “readers” (IGF2BP1, IGF2BP2, IGF2BB3, YTHDC2, YTHDF2, and YTHDF3) in both GC tissues and normal gastric mucosa tissues through the TCGA database. All six “readers” showed high expression in GC tissues (Figure 9M). Downregulation of the expression of these “readers” in GC cells revealed that only YTHDF2 affected the expression of TRIM50 mRNA (Figure 9N, S7F). A negative correlation between TRIM50 expression and YTHDF2 levels was observed in GC patients from the TCGA cohort (Figure 9O). Downregulation of YTHDF2 increased the protein levels of TRIM50 in GC cells (Figure 9P, S7G). RNA immunoprecipitation assays confirmed the direct interaction between the YTHDF2 protein and TRIM50 mRNA (Figure 9Q, 9R, S7H, S7I). Moreover, the actinomycin D experiment demonstrated that downregulation of YTHDF2 increased the stability of TRIM50 mRNA in GC cells (Figure 9S, S7J).

To identify specific m6A modification sites on TRIM50 mRNA, three m6A-Atlas databases (RMVar, RMBasev2.0 and SRAMP) were utilized. The intersection of these predictions revealed three potential modification sites (Figure 9T). chr7:73323916 and chr7:73327598 were identified as the major sites on TRIM50 mRNA through progressive mutagenesis of reporter plasmids (Figure 9U).

## Discussion

The tripartite motif (TRIM) domain-containing protein superfamily is widely acknowledged to be one of the largest monomeric E3 ubiquitin ligase families 18. The involvement of the TRIM family in the occurrence and development of cancer has been well established 19. In the context of GC, increasing amounts of evidence highlight the role of TRIM proteins. Specifically, TRIM59 is upregulated in gastric tumors, promoting the ubiquitination and degradation of p53, thereby facilitating GC development 20. Furthermore, TRIM32 activates the β-catenin signaling pathway in GC, resulting in enhanced cell proliferation and invasion 21. TRIM15 exerts antitumor effects by suppressing the invasion of GC cells 22. As a novel member of the TRIM family 6, TRIM50 may possess similar functions to other TRIM members due to its high homology 18. In this study, we observed a significant downregulation of TRIM50 in GC tissues, which correlated closely with tumor size, distant metastasis, and TNM staging. These findings align with previous research on TRIM50 in tumors 7, 8, 9, indicating a correlation between diminished TRIM50 expression and a poor prognosis in GC.

PGK1 is a critical enzyme in the glycolytic pathway whose role involves catalyzing the conversion of phosphoglycerate to 1,3-bisphosphoglycerate and generating ATP 23. Apart from its role in energy metabolism, recent research results have emphasized the importance of PGK1 in tumor development 24, 25, 26, 27. PGK1 is overexpressed in various cancer types and actively participates in essential cellular signaling pathways, including those related to PI3K/AKT, mTOR, and HIF-1α, promoting cancer cell proliferation, metastasis, and immune evasion 28, 29, 30. Furthermore, PGK1 plays a vital role in modulating the tumor microenvironment 30. Studies have revealed a strong correlation between PGK1 overexpression and increased levels of lactate produced by tumor cells 32. The accumulation of lactate leads to immune cell differentiation and activation, thereby promoting tumor progression 33. Therefore, targeting PGK1 and its associated molecular pathways involved in lactate metabolism represents a promising therapeutic strategy for cancer treatment 34. Previous research has shown that the E3 ligase STUB1 promotes the ubiquitination of the rate-limiting enzyme PGK1, thereby inhibiting the Warburg effect, tumor growth, and metastasis in breast cancer 35. In our study, we have made a novel discovery by demonstrating that TRIM50 can suppress the ubiquitination of PGK1, a finding that contrasts with previous studies and suggests variability in the E3 ubiquitin ligases targeting PGK1. We propose that this difference reflects the diverse adaptability of the structural and substrate binding sites of E3 ubiquitin ligases. Furthermore, in contrast to the prior study 9, we have confirmed that the impact of TRIM50 on the invasion and migration of GC cells is indirectly mediated by lactate-induced M2 polarization of TAMs, a mechanism that resembles our earlier study on GC cells promoting M2 polarization of macrophages through exosomes 17. However, this study highlighted the role of lactate in the crosstalk between tumor cells and TAMs, aligning with findings from several previous investigations 36, 37, 38. In summary, our findings elucidated a novel mechanism by which TRIM50 suppresses glycolysis by inhibiting the ubiquitination of PGK1, thereby impeding the proliferation and invasive migration of GC cells.

Moreover, previous research has indicated that K-48-linked polyubiquitination can induce the polyubiquitin degradation of target proteins 39, 40, while K-63-linked polyubiquitination primarily regulates the activation of target proteins 41, 42. In this study, we found that TRIM50 induced K-48-linked polyubiquitination of the PGK1 protein but not K-63-linked polyubiquitination. These findings align with those of previous research. Furthermore, in this study we identified K106 and K141 as the main sites of ubiquitination on the PGK1 protein. This result provided further molecular insights into the ubiquitination of PGK1 compared to previous studies.

The regulatory mechanisms of gene expression include transcription factor regulation, histone modifications, methylation, cytokines, and signal transduction, among others 43. Methylation, in particular, can be categorized into DNA methylation and RNA methylation 44. However, there have been limited reports on the regulatory mechanisms of the TRIM family, with only a few studies highlighting the role of m6A methylation in the regulation of TRIM family gene expression 45, 46, 47. METTL3/YTHDF2 has been shown to regulate TRIM7 through N6-methyladenosine modification, thereby participating in the tumor occurrence and chemotherapy resistance of osteosarcoma 45. METTL3/IGF2BP2, through the regulation of the N6-methyladenosine modification of TRIM11, is involved in the chemotherapy resistance of nasopharyngeal carcinoma 46. N6-methyladenosine modification of Trim59 mRNA, mediated by METTL3, is implicated in the prevention of acute respiratory distress syndrome induced by sepsis 47. In our study, we have made the novel discovery that the downregulation of TRIM50 in GC is regulated by m6A methylation, with the METTL3/YTHDF2 axis playing a crucial role in the regulation of m6A modification.

This study has several limitations that should be acknowledged. First, multiple E3 ubiquitin ligases with regulatory potential were identified by array sequencing and E3 ubiquitin ligase gene set expression analysis; however, for mechanistic analysis, we concentrated on TRIM50, which showed the greatest differential expression. Thus, the comprehensive role of other E3 ubiquitin ligases remains unknown and warrants further exploration. Second, the direct demonstration of the inhibitory effect of TRIM50 on GC metastasis was limited by the absence of an *in vivo* mouse model of orthotopic tumor metastasis in this study. Furthermore, the mechanism by which TRIM50 indirectly inhibits the invasion and migration of GC cells through lactate involves multiple factors. Apart from affecting the M2 polarization of TAMs, lactate can also play a role in regulating the activities of various other immune cells. It has been shown to influence the activation and function of Cancer-Associated Fibroblasts (CAFs), impairing their tumor-restricting potential 48. Additionally, lactate can dampen the cytotoxic activity of Natural Killer (NK) cells and Cytotoxic T-lymphocytes by disrupting their metabolic programs, thereby attenuating their capacity to eliminate cancer cells 49, 50. Furthermore, lactate impacts Dendritic Cell maturation and antigen presentation, which are critical for initiating effective antitumor immune responses 51. Lactate also plays a role in the polarization of Neutrophils to an N2 phenotype, associated with oncogenic functions such as the promotion of angiogenesis, immunosuppression, and facilitation of metastatic processes 52. Collectively, these effects of lactate on immune cells can significantly influence the metastatic potential of tumors by creating an immunosuppressive microenvironment that favors tumor cell survival, invasion, and the formation of distant metastases. However, the involvement of these additional cell types and the specific mechanisms by which TRIM50 affects their function were not thoroughly investigated in this study. Moreover, the lactate-trained M2 polarization of TAMs involves multiple mechanisms 36, 37, 38. Further research is needed to elucidate the molecular pathways underlying lactate-trained M2 polarization and the specific contributions of TRIM50 to this process. Addressing these limitations and conducting more comprehensive studies will contribute to a better understanding of the role of TRIM50 in GC and its regulation within the tumor microenvironment, as well as its potential therapeutic implications.

## Conclusion

In this study, we definitively identified TRIM50 as a TSG that effectively inhibits aerobic glycolysis and malignant behavior in GC. Functionally, the overexpression of TRIM50 attenuated the proliferative capacity of cancer cells and indirectly influenced the invasive and migratory abilities of GC cells by suppressing the M2 polarization of TAMs. Mechanistically, TRIM50 inhibited the glycolytic pathway of GC cells by ubiquitinating and degrading PGK1, thereby directly suppressing GC cell proliferation. Simultaneously, the reduction in lactate led to diminished M2 polarization of TAMs, indirectly inhibiting the invasion and migration of GC cells. Notably, the downregulation of TRIM50 in GC was mediated by the METTL3/YTHDF2 axis in an m6A-dependent manner. Importantly, these findings suggest that TRIM50 could serve as a potential novel therapeutic target for the treatment of malignant tumors.

## Supplementary Material

Supplementary figures, tables, and methods.

## Figures and Tables

**Figure 10:**
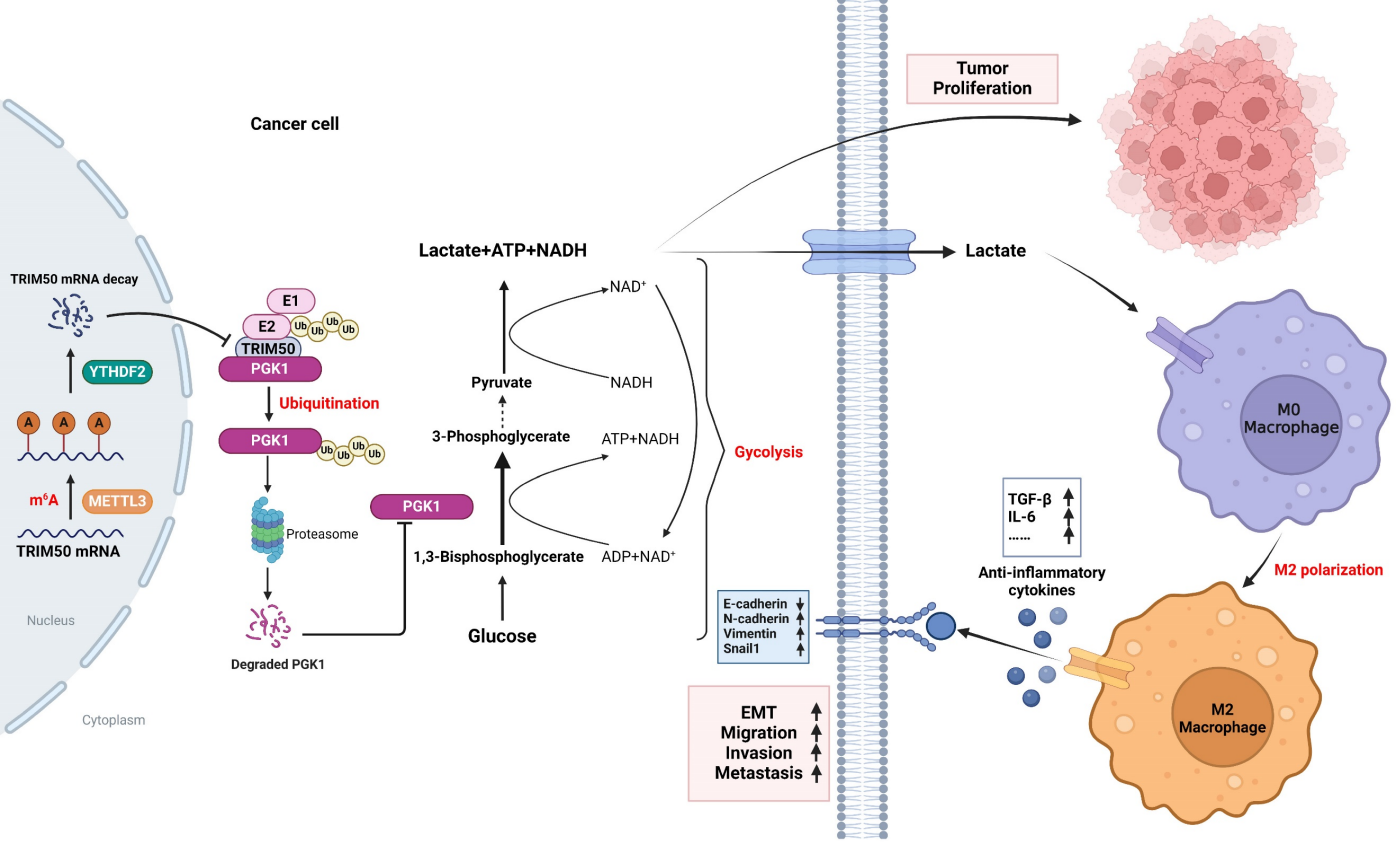
** Mechanistic overview of TRIM50's regulation of the glycolytic pathway and its role in inhibiting the malignant phenotype of GC cells through PGK1 ubiquitination.** TRIM50 mediates the degradation of lactate dehydrogenase PGK1, thereby suppressing the glycolytic pathway in GC and directly inhibiting GC cell proliferation. Simultaneously, the reduction of lactate, a glycolytic product, leads to decreased infiltration of TAMs and a decrease in M2 polarization in the tumor microenvironment. This, in turn, results in reduced secretion of EMT-related cytokines, such as TGF-β and IL-6, indirectly inhibiting the invasive and migratory abilities of GC cells. The low expression of TRIM50 in GC cells is mainly mediated by mRNA m6A methylation.

**Table 1:** The correlation between TRIM50 mRNA expression and the patients' clinical pathology data

Characteristic	Overall	Expression of TRIM50		p
		High	Low	
N	124	62	62	
Age, year	64.92 ± 11.70	63.53 ± 12.38	66.31 ± 10.91	0.188
Gender, n (%)				0.208
Female	59 (47.6%)	26 (21.0%)	33 (26.6%)	
Male	65 (52.4%)	36 (29.0%)	29 (23.4%)	
Tumor volume, cm^3^	17.86 (13.64, 26.76)	15.75 (13.13, 20.30)	23.65 (14.11, 29.13)	**0.002**
T classification, n (%)				0.170
I	8 (6.5%)	6 (4.8%)	2 (1.6%)	
II	21 (16.9%)	12 (9.7%)	9 (7.3%)	
III	40 (32.3%)	22 (17.7%)	18 (14.5%)	
IV	55 (44.4%)	22 (17.7%)	33 (26.6%)	
N classification, n (%)				0.407
0	25 (20.2%)	16 (12.9%)	9 (7.3%)	
1	25 (20.2%)	12 (9.7%)	13 (10.5%)	
2	40 (32.3%)	17 (13.7%)	23 (18.5%)	
3	34 (27.4%)	17 (13.7%)	17 (13.7%)	
M classification, n (%)				**0.011**
0	116 (93.5%)	62 (50.0%)	54 (43.5%)	
1	8* (6.5%)	0 (0%)	8* (6.5%)	
Pathologic stage, n (%)				**0.011**
I	12 (9.7%)	8 (6.5%)	4 (3.2%)	
II	40 (32.3%)	24 (19.4%)	16 (12.9%)	
III	64 (51.6%)	30 (24.2%)	34 (27.4%)	
IV	8* (6.5%)	0 (0%)	8* (6.5%)	
OS, month	18.93 (12.02, 34.24)	28.05 (13.38, 36.92)	14.30 (10.42, 32.12)	**0.004**

“*” denotes patients with resectable single distant metastasis. Bold values indicate an intergroup p <0.05, indicating the presence of a statistically significant difference.

## Abbreviations

ATP: adenosine triphosphate
CCK8: cell counting kit-8
Co-IP: Coimmunoprecipitation
ECAR: Extracellular acidification rate
ELISA: enzyme-linked immunosorbent assay
EMT: Epithelial-mesenchymal transition
GC: gastric cancer
GSEA: gene set enrichment analysis
H&E: hematoxylin and eosin
IHC: immunohistochemistry
IVIS: *In vivo* Imaging System
m6A: N6-methyladenosine
MeRIP-seq: methylated RNA-immunoprecipitation sequencing
OCR: Oxygen consumption rate
OS: overall survival
OXPHOS: Oxidative phosphorylation
PGK1: Phosphoglycerate Kinase 1
PMA: phorbol 12-myristate 13-acetate
qRT‒PCR: quantitative real-time polymerase chain reaction
RNA-Seq: RNA sequencing
ROS: Reactive Oxygen Species
siRNA: small interfering RNA
TGF-β: Transforming Growth Factor Beta
TME: Tumor microenvironment
TRIM50: Tripartite motif-containing protein 50
TSG: Tumor suppressor gene
UPS: ubiquitin-proteasome system
