# Supplementary material for: TRIM50 inhibits glycolysis and the malignant progression of gastric cancer by ubiquitinating PGK1
Source: Int J Biol Sci. 2024 Jul 1;20(9):3656–74. doi: 10.7150/ijbs.97091 (PMC11234210; doi:10.7150/ijbs.97091)
Supplement: Supplementary file 1 — Supplementary figures, tables, and methods. [file ijbsv20p3656s1.pdf]

Supplementary figures

Supplementary Figure 1

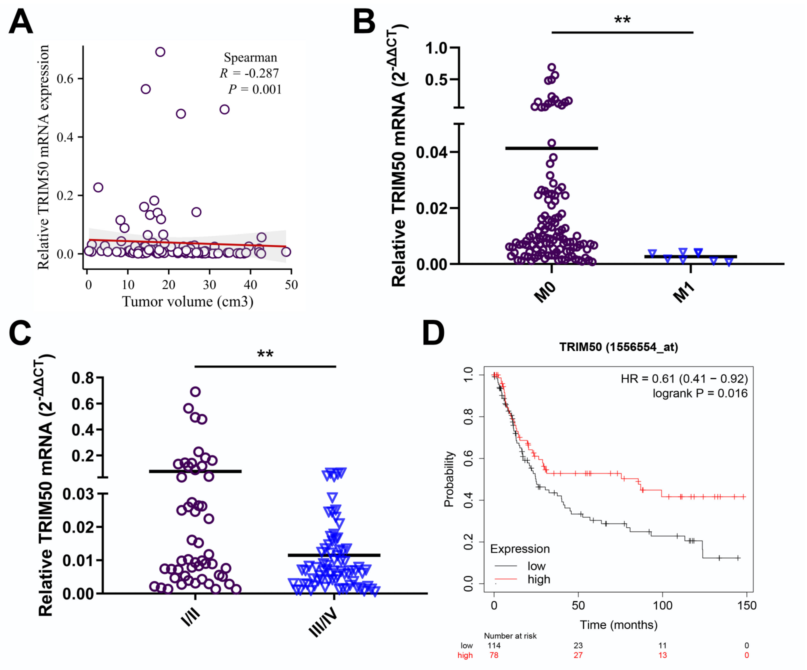

Figure S1. (A) Correlation between TRIM50 mRNA expression and tumor volume in 60 pairs of GC tissues, calculated by Spearman correlation analysis. **(B)** Correlation between TRIM50 mRNA expression and distant metastasis. **(C)** Correlation between TRIM50 mRNA expression and TNM staging. **(D)** Survival analysis of GC patients in GSE15459 dataset based on TRIM50 mRNA expression. The data are representative of three independent experiments. Quantitative data are shown as mean ± SD. *P < 0.05, **P < 0.01, ***P < 0.001 (Student’s t-test).

Supplementary Figure 2

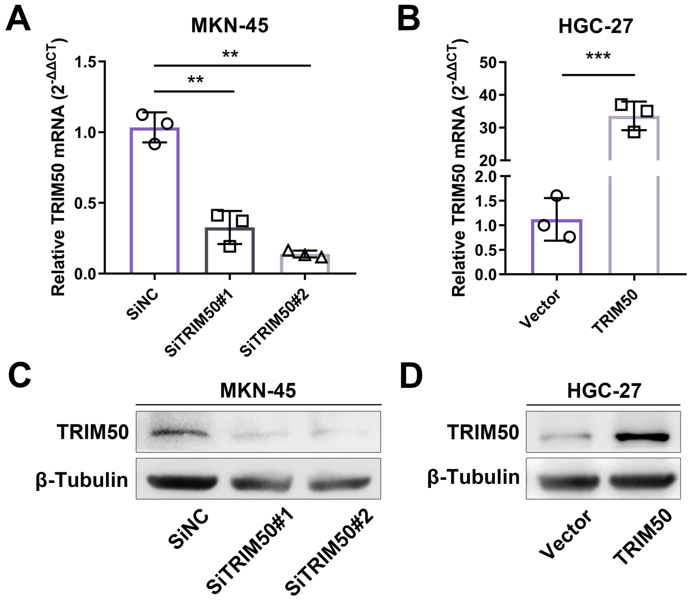

Figure S2. (A), **(B)** qRT–PCR analysis demonstrated the expression of TRIM50 mRNA in GC cells MKN-45 or HGC-27 after transfection with siRNAs (A) or overexpression plasmids (B). **(C)**, **(D)** Western blotting analysis showed the expression of TRIM50 protein in GC cells MKN-45 or HGC-27 after transfection with siRNAs (C) or overexpression plasmids (D). The data are representative of three independent experiments. Quantitative data are shown as mean ± SD. $*P < 0.05$, $**P < 0.01$, $***P < 0.001$ (Student's t-test).

Supplementary Figure 3

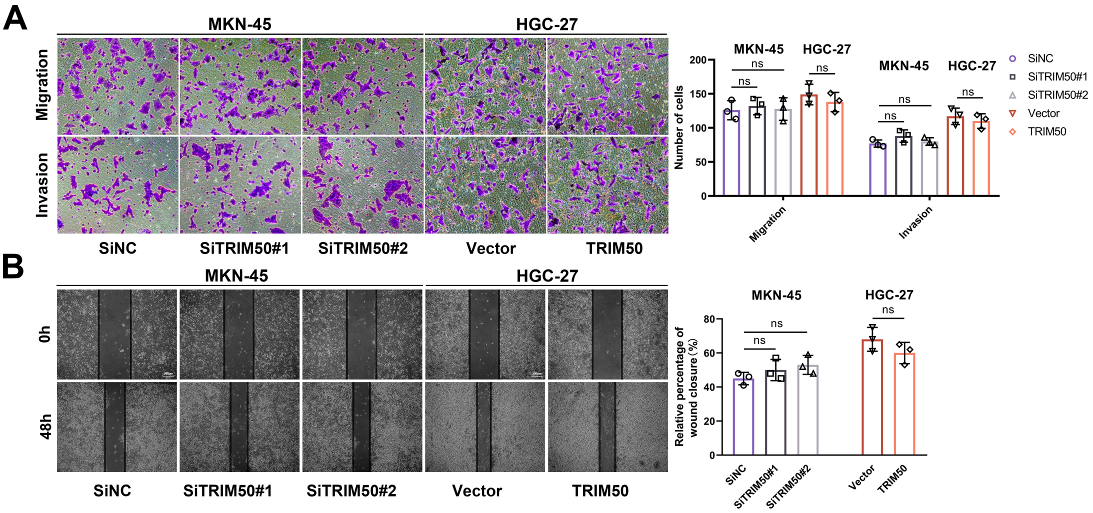

Figure S3. (A) Transwell assay demonstrated the effect of TRIM50 on the invasion and migration of GC cells. **(B)** Wound healing assay showed the effect of TRIM50 on the invasion and migration of GC cells. The data are representative of three independent experiments. Quantitative data are shown as mean ± SD. *P < 0.05, **P < 0.01, ***P < 0.001 (Student's t-test).

Supplementary Figure 4

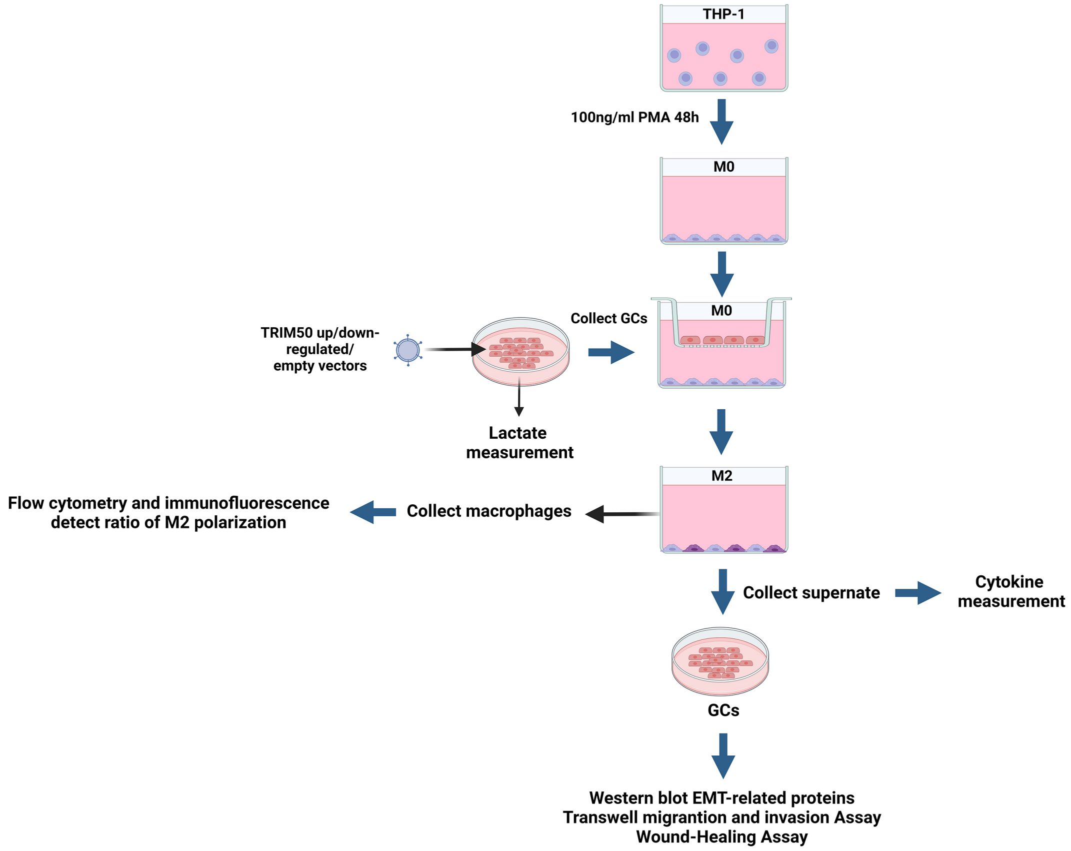

Figure S4. Schematic representation of the establishment process of the coculture model between GC cells and M0 macrophages.

Supplementary Figure 5

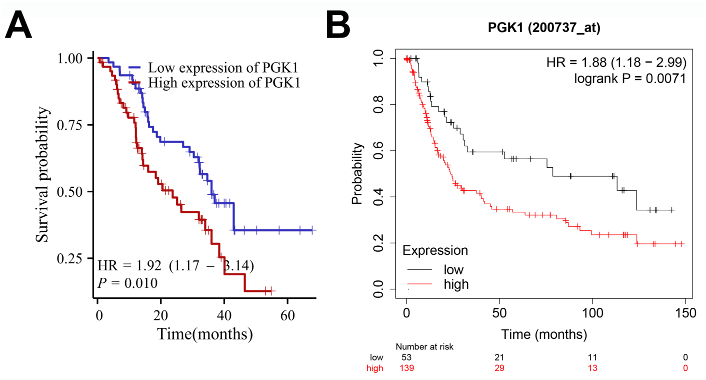

Figure S5 (A) Survival analysis of 124 GC patients based on PGK1 mRNA expression. **(B)** Survival analysis of GC patients in GSE15459 dataset based on PGK1 mRNA expression. The median expression level of PGK1 was used as the cut-off value to classify patients into high and low expression groups.

Supplementary Figure 6

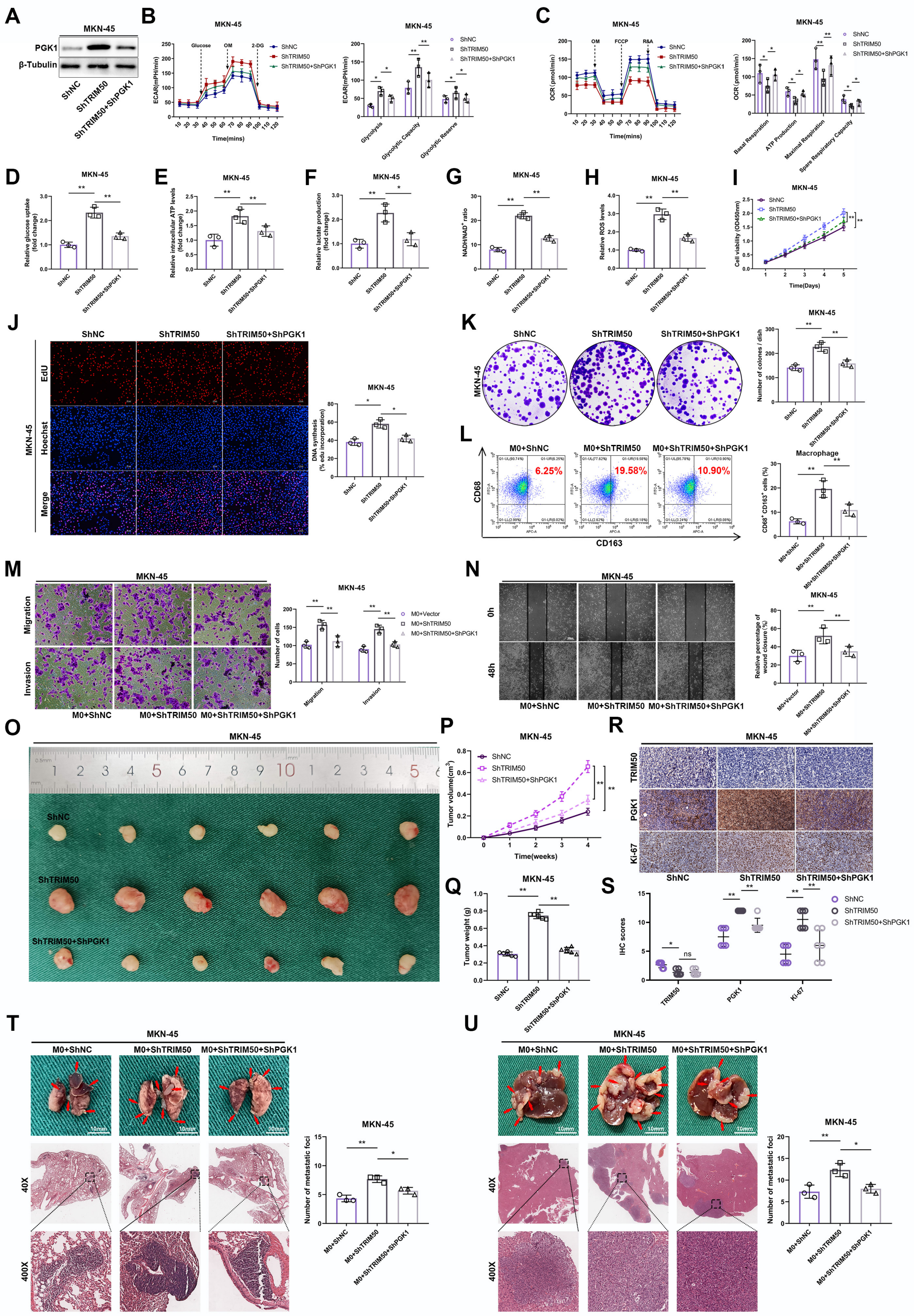

Figure S6. (A) Western blotting analysis of PGK1 expression in MKN-45-ShTRIM50 cells. **(B)** ECAR assay demonstrated that silencing PGK1 reversed the enhanced glycolytic capacity caused by TRIM50 knockdown in MKN-45 cells. **(C)** OCR assay showed that silencing PGK1 reverses the inhibitory effect of TRIM50 knockdown on mitochondrial oxidative phosphorylation capacity in MKN-45 cells. **(D)-(H)** Silencing PGK1 expression reversed the enhanced effects of TRIM50 knockdown on glucose uptake (D), ATP levels (E), lactate secretion (F), NADH/NAD+

conversion (G), and ROS levels (H) in MKN-45 cells. **(I)** CCK-8 assay, **(J)** EdU assay, and **(K)** colony formation assay demonstrated that silencing PGK1 expression reverses the enhanced proliferative capacity caused by decreased TRIM50 in MKN-45 cells. Scale bar: 100 μm (J). **(L)** Flow cytometry analysis showed that silencing PGK1 expression reversed the M2 polarization degree of macrophages cocultured with MKN-45 cells with decreased TRIM50. **(M)**, **(N)** Transwell (M) and wound healing assay (N) demonstrated that silencing PGK1 expression reverses the indirect enhancement of decreased TRIM50 on invasion and migration ability of GC cells cultured with macrophage supernatant. Scale bar: 100 μm (M), 200 μm (N). **(O)** The mouse model of subcutaneous xenograft tumor was used to evaluate the effect of inhibiting PGK1 expression on reversing the indirect promotion of tumor growth caused by TRIM50 knockdown ($n = 6$). **(P)** Time–volume curve of subcutaneous xenograft tumors. **(Q)** Weight of subcutaneous xenograft tumors. **(R)** Immunohistochemical staining of TRIM50, PGK1, and Ki-67 in subcutaneous xenograft tumorswas performed, with **(S)** showing the quantified staining scores. **(T)**, **(U)** Mouse models of lung (T) and liver metastasis (U) were used to evaluate how PGK1 inhibition reversed the indirect promotional effects of TRIM50 on metastasis, with H&E staining for evaluation ($n=3$ for each). The data are representative of three independent experiments. p values were determined by two-way ANOVA test (**I**, **P**), or two-tailed unpaired Student's t test (**B**, **C**, **D**, **E**, **F**, **G**, **H**, **J**, **K**, **L**, **M**, **N**, **Q**, **S**, **T**, **U**) (*$P < 0.05$, **$P < 0.01$, ***$P < 0.001$)

Supplementary Figure 7

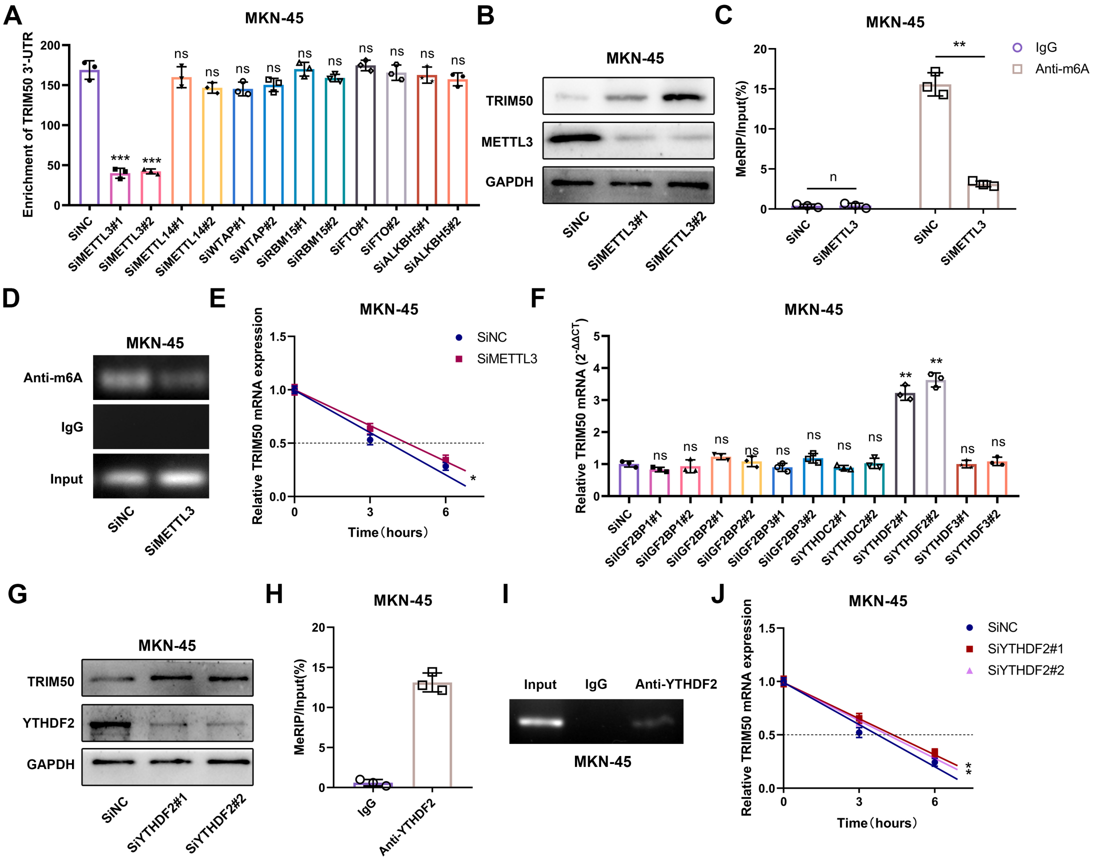

Figure S7. (A) Expression of m6A writers (methyltransferases) METTL3, METTL14, METTL16, RBM15, WATP, VIRMA, and erasers (demethylases) FTO and ALKBH5 was knocked down in GC cell line (MKN-45). The m6A levels in the TRIM50 3'-UTR were measured by qRT–PCR, showing the most significant decrease in m6A levels after knockdown of METTL3. **(B)** Western blot analysis of TRIM50 expression in GC cells (MKN-45) following knockdown of METTL3. **(C)** qRT–PCR and **(D)** agarose gel electrophoresis analysis demonstrated changes in m6A levels in the TRIM50 3'-UTR using MeRIP after METTL3 knockdown in MKN-45 cells. **(E)** TRIM50 mRNA stability was assessed using the actinomycin D experiment in MKN-45 cells with METTL3 knockdown. **(F)** The effect of knockdown of stability-related m6A readers (IGF2BP1, IGF2BP2, IGF2BB3, YTHDC2, YTHDF2, and YTHDF3) on TRIM50 mRNA expression was observed by knocking down their expression in GC cell line (HGC-27). **(G)** Western blotting analysis of TRIM50 expression in GC cells (HGC-27) following knockdown of METTL3. **(H)** qRT–PCR and **(I)** agarose gel electrophoresis analysis demonstrated changes in m6A levels in the TRIM50 3'-UTR using MeRIP after METTL3 knockdown in GC cells (HGC-27). **(J)** TRIM50 mRNA stability was evaluated using the actinomycin D experiment in GC cells (HGC-27) with METTL3 knockdown. The data are representative of three independent experiments. Quantitative data are shown as mean ± SEM (**E**, **J**), or mean ± SD (**A**, **C**, **F**, **H**). p values were determined by two-way ANOVA test (**E**, **J**), two-tailed unpaired Student's t test (**A**, **C**, **F**, **H**) (*P < 0.05, **P < 0.01, ***P < 0.001).

Supplementary tables

Supplementary Table S1

Clinical information of 24 patients for RNA sequencing

Sample number	Gender	Age(year)	TNM Stage
S1-RE,S2-RE	Male	71	T4bN0M0(IIIA)
S3,S4	Male	64	T2N0M0(IB)
S5,S6	Female	60	T2N0M0(IB)
S7,S8	Male	65	T2N0M0(IB)
S9,S10	Male	76	T2N0M0(IB)
S11-RE,S12-RE	Female	50	T4aN0M0(IIB)
S13,S14	Male	58	T1N0M0(IA)
S15,S16	Male	78	T1N0M0(IA)
S17,S18	Female	73	T1N1M0(IB)
S19,S20	Male	51	T1N0M0(IA)
S21,22	Female	63	T1N0M0(IA)
S23,24	Male	64	T1N0M0(IA)
SS25,S26	Female	67	T4aN2M0(IIIA)
S27,S28	Male	62	T4aN3bM0(IIIC)
S29,S30	Male	58	T4aN3bM0(IIIC)
S31,S32	Female	63	T4aN3bM0(IIIC)
S33,S34	Male	66	T4aN3aM0(IIIB)
S35,36	Male	75	T4aN3aM0(IIIB)
S37,S38	Female	72	T4aN2M0(IIIA)
S39,S40	Male	65	T4aN2M0(IIIA)
S41,S42	Male	66	T4aN3aM0(IIIB)
S43,S44	Male	67	T4aN3aM0(IIIB)
S45,S46	Female	60	T4aN3aM0(IIIB)
S47,S48	Male	66	T4aN3aM0(IIIB)

Note: Sample numbers with odd numbers represent tumor tissues; sample numbers with even numbers represent normal paracancerous tissue

Supplementary Table S2

Clinical information of 124 patients for PCR and prognostic analysis

Patient number	Gende r	Age (year)	Tumor volume(cm^3)	TNM Stage	Relative TRIM50 mRNA($2^{-\triangle\triangle CT}$)		Relative PGK1 mRNA($2^{-\triangle\triangle CT}$)		Status	OS (month)
					Tumor	Normal	Tumor	Normal		
1	Male	59	17.93	T1N0M0(IA)	0.691	0.460	5.129	4.269	Alive	29.13
2	Female	58	14.39	T3N1M0(IIB)	0.564	0.379	5.384	3.528	Dead	15.80
3	Female	54	33.66	T3N0M0(IIA)	0.494	0.271	5.671	5.704	Alive	31.63
4	Male	65	22.99	T3N0M0(IIA)	0.479	0.378	6.258	5.643	Alive	34.10
5	Female	37	2.77	T1N1M0(IA)	0.227	1.179	5.582	3.269	Alive	63.93
6	Male	68	16.47	T1N1M0(IB)	0.182	0.843	5.987	3.418	Dead	18.73
7	Female	74	13.99	T4aN0M0(IIB)	0.161	1.617	6.125	5.693	Dead	19.13
8	Male	78	26.77	T2N1M0(IIA)	0.143	0.365	5.743	5.652	Alive	37.47
9	Female	51	17.27	T3N0M0(IIA)	0.141	0.779	5.789	5.879	Alive	13.33
10	Female	42	15.43	T3N0M0(IIA)	0.133	1.331	6.036	5.649	Alive	22.50
11	Male	68	18.12	T3N1M0(IIB)	0.118	1.278	7.389	3.645	Dead	6.40
12	Male	63	8.19	T3N0M0(IIA)	0.115	1.338	5.495	4.259	Alive	43.13
13	Female	61	9.20	T1N2M0(IIA)	0.088	1.235	6.753	3.628	Alive	53.00
14	Male	69	18.66	T4aN3aM0(IIIB)	0.066	1.341	5.612	4.721	Dead	19.80
15	Female	72	14.86	T3N2M0(IIIA)	0.064	1.678	7.621	3.987	Alive	21.47
16	Female	69	42.70	T4aN3aM0(IIIB)	0.057	2.398	5.379	6.185	Dead	29.07
17	Male1	70	8.40	T4aN3aM0(IIIB)	0.043	0.852	6.548	6.237	Alive	39.00
18	Female	74	16.08	T3N1M0(IIB)	0.038	1.439	5.847	5.749	Dead	17.53
19	Male	78	19.00	T3N2M0(IIIA)	0.036	0.480	7.195	4.721	Dead	1.73
20	Female	73	1.20	T4aN0M0(IIB)	0.032	0.843	5.632	3.875	Alive	43.33
21	Female	83	26.30	T2N3aM0(IIIA)	0.029	1.000	6.489	3.718	Dead	25.13
22	Female	29	4.40	T1N1M0(IA)	0.027	0.543	5.926	3.549	Alive	50.23
23	Male	67	4.34	T2N0M0(IB)	0.026	0.866	5.751	3.145	Alive	67.73
24	Male	66	25.40	T4aN0M0(IIB)	0.026	1.617	5.652	6.314	Alive	36.16
25	Male	57	11.80	T3N0M0(IIA)	0.026	1.405	7.854	3.987	Alive	12.60
26	Female	57	23.60	T2N1M0(IIA)	0.025	1.175	7.712	5.749	Alive	33.13
27	Male	71	39.60	T3N3aM0(IIIB)	0.025	0.659	5.864	3.697	Dead	16.26
28	Female	61	14.27	T3N0M0(IIA)	0.025	0.358	5.623	4.583	Alive	12.93
29	Male	80	31.20	T3N3aM0(IIIC)	0.025	0.681	6.398	3.544	Dead	12.23
30	Male	66	20.55	T3N2M0(IIIA)	0.025	1.152	6.579	4.265	Dead	4.60
31	Female	76	19.12	T4bN1M0(IIIB)	0.023	0.878	7.643	5.938	Alive	8.93
32	Male	74	13.80	T1N1M0(IA)	0.022	0.939	5.652	4.357	Alive	57.33
33	Female	72	16.12	T3N2M0(IIIA)	0.021	1.413	6.439	3.786	Dead	38.43
34	Male	55	42.10	T4aN2M0(IIIA)	0.018	0.717	5.712	4.364	Alive	38.33
35	Female	47	20.50	T2N3aM0(IIIA)	0.017	1.038	5.905	4.892	Alive	36.67
36	Female	41	25.29	T3N2M0(IIIA)	0.017	1.000	7.582	6.043	Alive	12.23
37	Female	49	14.62	T4aN0M0(IIB)	0.016	1.262	5.938	4.573	Alive	32.97

38	Female	67	14.10	T3N3aM0(IIIB)	0.016	1.005	6.591	5.879	Alive	26.20
39	Male	63	15.64	T4aN1M0(IIIA)	0.016	1.177	7.431	4.582	Dead	18.40
40	Male	77	16.82	T2N2M0(IIB)	0.015	0.424	5.697	4.625	Dead	14.27
41	Male	43	15.70	T4aN3aM0(IIIB)	0.015	1.000	6.543	5.015	Dead	9.70
42	Male	59	13.80	T4aN3aM0(IIIB)	0.013	0.699	6.492	4.361	Dead	32.03
43	Male	84	17.91	T3N2M0(IIIA)	0.013	0.826	5.884	4.512	Dead	13.53
44	Male	63	15.16	T3N2M0(IIIA)	0.013	1.175	7.369	5.649	Dead	5.53
45	Male	59	17.68	T3N3bM0(IIIC)	0.013	1.176	5.635	3.544	Dead	3.50
46	Female	47	15.40	T4aN2M0(IIIA)	0.012	0.641	5.931	5.473	Alive	40.53
47	Male	53	10.50	T4aN2M0(IIIA)	0.012	0.972	5.672	3.584	Alive	34.67
48	Male	85	24.29	T2N0M0(IB)	0.012	1.577	7.683	4.583	Alive	5.60
49	Male	61	15.80	T4aN2M0(IIIA)	0.011	0.777	6.349	3.528	Alive	32.67
50	Male	57	3.55	T3N1M0(IIB)	0.010	0.641	6.548	4.758	Alive	54.87
51	Male	66	19.70	T4aN0M0(IIB)	0.010	0.478	5.896	4.581	Alive	41.73
52	Female	74	28.90	T4aN2M0(IIIA)	0.010	0.365	7.683	3.321	Dead	40.10
53	Male	79	0.50	T4aN3aM0(IIIB)	0.010	0.540	7.145	5.841	Alive	32.83
54	Female	45	0.50	T4aN3aM0(IIIB)	0.010	0.713	5.945	3.645	Dead	27.03
55	Female	61	13.66	T3N1M0(IIB)	0.010	0.540	6.499	3.544	Alive	20.23
56	Male	86	10.00	T2N2M0(IIB)	0.009	0.271	5.896	3.517	Dead	37.00
57	Male	66	12.80	T2N1M0(IIA)	0.009	0.460	5.712	4.582	Alive	33.20
58	Male	51	13.60	T4aN2M0(IIIA)	0.009	0.379	5.672	4.704	Dead	32.33
59	Female	73	12.98	T3N2M0(IIIA)	0.009	1.372	6.559	5.649	Alive	12.70
60	Male	60	12.62	T3N0M0(IIA)	0.009	0.478	7.523	4.265	Dead	12.57
61	Male	65	13.80	T4aN2M0(IIIA)	0.008	1.283	6.579	5.187	Dead	46.50
62	Female	64	28.90	T1N1M0(IA)	0.008	0.378	5.733	4.965	Alive	40.00
63	Male	41	12.30	T1N1M0(IA)	0.008	1.341	5.925	3.697	Alive	33.10
64	Male	57	17.80	T4aN2M0(IIIA)	0.008	0.575	5.876	5.938	Alive	32.00
65	Female	73	11.50	T4aN3aM0(IIIB)	0.008	0.815	6.579	3.749	Dead	14.40
66	Male	69	28.66	T3N0M0(IIA)	0.008	1.399	6.419	3.685	Alive	9.33
67	Male	51	5.10	T3N2M0(IIIA)	0.007	1.273	5.644	5.964	Alive	46.30
68	Male	50	26.20	T3N2M0(IIIA)	0.007	0.571	5.785	6.147	Dead	43.00
69	Male	75	16.70	T4aN2M0(IIIA)	0.007	1.338	5.712	5.829	Dead	36.00
70	Female	63	7.28	T4aN2M0(IIIA)	0.007	0.865	6.349	3.947	Dead	36.00
71	Female	62	18.20	T4aN1M0(IIIA)	0.007	0.358	6.492	3.418	Dead	34.00
72	Female	74	1.10	T2N0M0(IB)	0.007	0.364	5.885	3.685	Alive	32.16
73	Female	70	23.66	T2N2M0(IIB)	0.007	0.795	7.399	6.043	Dead	20.53
74	Female	70	23.65	T4aN2M0(IIIA)	0.007	0.381	5.931	5.938	Dead	14.50
75	Male	71	29.20	T4aN2M0(IIIA)	0.007	0.981	6.538	4.269	Dead	14.00
76	Male	77	18.31	T1N1M0(IB)	0.007	0.881	7.268	4.512	Alive	6.30
77	Female	70	23.97	T2N2M0(IIB)	0.007	0.717	7.523	5.879	Dead	6.17
78	Female	68	48.71	T3N2M0(IIIA)	0.007	0.571	7.337	6.043	Dead	4.07
79	Male	77	11.50	T2N1M0(IIA)	0.006	1.562	5.784	4.352	Alive	36.03
80	Female	80	42.10	T4aN3aM0(IIIB)	0.006	0.762	5.945	6.015	Dead	32.26
81	Female	49	27.40	T4aN3aM0(IIIB)	0.006	0.475	6.499	4.625	Dead	32.00

82	Male	69	20.90	T4aN2M0(IIIA)	0.006	0.762	5.652	3.645	Alive	13.87
83	Female	70	22.24	T3N2M0 (IIIA)	0.006	0.965	7.205	5.693	Alive	1.67
84	Male	80	11.50	T2N1M0(IIA)	0.005	0.424	5.691	3.698	Dead	43.00
85	Male	66	8.30	T3N1M0(IIB)	0.005	0.566	5.914	3.675	Alive	36.13
86	Female	56	10.50	T3N2M0(IIIA)	0.005	0.269	5.896	4.216	Dead	36.00
87	Male	75	14.02	T4aN3aM0(IIIB)	0.005	0.381	5.905	5.749	Alive	11.93
88	Female	90	26.75	T2N1M0(IIB)	0.005	1.438	7.208	3.418	Alive	1.37
89	Male	60	12.80	T4aN2M0(IIIA)	0.004	0.549	5.691	4.761	Dead	34.67
90	Male	71	18.40	T4aN1M0(IIIA)	0.004	0.480	5.925	3.749	Dead	32.00
91	Female	46	16.95	T3N2M0(IIIA)	0.004	0.269	6.419	4.512	Dead	26.47
92	Female	66	27.82	T2N2M0(IIB)	0.004	0.364	5.876	4.265	Alive	21.20
93	Female	77	34.40	T4aN1M0(IIIA)	0.004	0.678	6.538	5.841	Dead	16.00
94	Female	58	34.80	T4bN2M1(IV)	0.004	0.973	5.733	4.582	Dead	12.00
95	Female	72	25.29	T3N3bM1(IV)	0.004	0.659	7.461	3.749	Dead	8.17
96	Male	58	14.62	T3N1M0(IIB)	0.004	0.279	7.399	4.625	Alive	7.50
97	Male	55	38.92	T4N3M1(IV)	0.004	1.000	7.461	4.582	Dead	0.70
98	Male	51	24.20	T2N0M0(IB)	0.003	1.077	6.492	6.097	Alive	35.10
99	Male	68	29.70	T4aN0M0(IIB)	0.003	1.415	5.885	3.895	Dead	30.40
100	Male	76	14.38	T4aN1M0(IIIA)	0.003	0.775	6.349	4.583	Dead	23.73
101	Male	70	11.20	T4aN0M0(IIB)	0.003	1.276	5.785	4.573	Dead	16.00
102	Female	46	18.90	T2N2M0(IIB)	0.003	0.778	5.931	6.015	Alive	15.00
103	Male	71	37.30	T3N3aM0(IIIB)	0.003	1.278	7.268	5.704	Dead	14.20
104	Male	69	28.90	T4bN3aM0(IIIC)	0.003	0.676	7.337	3.418	Dead	12.03
105	Female	67	27.20	T4bN3aM0(IIIC)	0.003	1.000	5.644	3.895	Dead	7.00
106	Female	60	13.36	T4aN2M0(IIIA)	0.002	1.283	5.896	3.685	Dead	14.97
107	Male	75	17.40	T4aN0M0(IIB)	0.002	0.279	7.683	6.185	Alive	14.00
108	Female	85	24.20	T4aN0M0(IIB)	0.002	1.085	6.559	4.721	Dead	12.13
109	Female	75	22.26	T3N2M0(IIIA)	0.002	0.949	7.205	3.697	Dead	11.60
110	Male	74	17.14	T4aN1M1(IV)	0.002	1.154	5.945	5.841	Dead	11.17
111	Female	58	36.80	T4bN3bM0(IIIC)	0.002	0.879	6.499	4.269	Dead	7.00
112	Male	74	32.10	T4bN3bM0(IIIC)	0.002	1.081	7.472	3.718	Dead	6.00
113	Male	51	28.20	T4aN0M0(IIB)	0.001	0.984	5.876	4.704	Alive	15.13
114	Male	59	25.90	T4aN0M0(IIB)	0.001	0.584	6.419	4.361	Alive	14.63
115	Female	62	38.40	T4aN3aM0(IIIB)	0.001	0.586	5.733	3.895	Dead	14.13
116	Female	58	30.60	T3N3aM0(IIIB)	0.001	1.281	7.523	3.718	Alive	13.13
117	Female	74	31.50	T3N3bM0(IIIC)	0.001	0.679	7.461	5.693	Dead	12.13
118	Female	72	30.77	T3N2M0(IIIA)	0.001	0.943	7.399	4.573	Alive	11.47
119	Male	82	28.90	T4aN1M0(IIIA)	0.001	0.582	5.905	3.528	Dead	11.00
120	Female	74	13.60	T4aN0M0(IIB)	0.001	1.184	5.672	4.704	Alive	10.23
121	Female	62	17.43	T3N3aM1(IV)	0.001	0.582	6.559	4.361	Dead	9.10
122	Male	58	42.59	T4bN3aM1(IV)	0.001	2.398	5.691	5.704	Dead	7.07
123	Female	81	37.49	T3N3bM1(IV)	0.001	0.584	6.579	5.185	Dead	6.67
124	Female	40	12.50	T3N2M0(IIIA)	0.001	1.087	5.925	3.987	Dead	5.03

Supplementary Table S3

The information of cell lines used in this study

GC cell lines	Species	Source	Morphology	Culture properties
GES-1	Human	Fetal gastric mucosa from a 9-month-old fetus	Epithelioid	Adherent growth
MKN45	Human	Metastatic lesion (liver) from a 62-year-old female, low-differentiated gastric adenocarcinoma patient	Epithelioid	Adherent and suspension growth
AGS	Human	Gastric adenocarcinoma tissues from a 54-year-old female patient who didn’t receive any preoperative treatment	Epithelioid	Adherent growth
HGC27	Human	Undifferentiated GC tissues	Epithelioid	Adherent growth
THP-1	Human	Peripheral blood from a one-year-old boy	Spheroid	Suspension growth

Supplementary Table S4

Primers used in the experiments

**Primer**		**Sequence 5'-3'**
β-Tubulin	Forward	TGGACTCTGTTCGCTCAGGT
	Reverse	TGCCTCCTTCCGTACCACAT
GAPDH	Forward	GGAGCGAGATCCCTCCAAAAT
	Reverse	GGCTGTTGTCATACTTCTCATGG
TRIM50	Forward	GGCCCTTAGAAGGCGCATT
	Reverse	GCAGGGTCCAACTTGAGAGG
PGK1	Forward	CCACTGTGGCTTCTGGCATA
	Reverse	ATGAGAGCTTTGGTTCCCCG
TGF-β	Forward	GGCCAGATCCTGTCCAAGC
	Reverse	GTGGGTTTCCACCATTAGCAC
IL-6	Forward	ATCAGAACACTGATCCAGATCC
	Reverse	CAAGGTTTCTCAGGATGAGG
METTL3	Forward	TTGTCTCCAACCTTCCGTAGT
	Reverse	CCAGATCAGAGAGGTGGTGTAG
METTL14	Forward	AGTGCCGACAGCATTGGTG
	Reverse	GGAGCAGAGGTATCATAGGAAGC
WTAP	Forward	CTTCCCAAGAAGGTTCGATTGA
	Reverse	TCAGACTCTCTTAGGCCAGTTAC
RBM15	Forward	ATGGAGGAGGTGGAGCGGG
	Reverse	TCAGCCCTTCCTGTTTCTGC
FTO	Forward	ACTTGGCTCCCTTATCTGACC
	Reverse	TGTGCAGTGTGAGAAAGGCTT
ALKBH5	Forward	CGGCGAAGGCTACACTTACG
	Reverse	CCACCAGCTTTTGGATCACCA
IGF2BP1	Forward	ATGGCTGCTGCTGCTGCTG
	Reverse	AGCAGCAGCAGCAGCCAT
IGF2BP2	Forward	ATGGCGGCGGCTGGGAACT
	Reverse	TCACACTTGTTCAAAGGTGCT
IGF2BP3	Forward	ATGAGGAGGAAAGGGAAAGG
	Reverse	CTATGTGTGGCCTTCCTCGT
YTHDC2	Forward	CAAAACATGCTGTTAGGAGCCT
	Reverse	CCACTTGTCTTGCTCATTTCCC
YTHDF2	Forward	ACACATTCGCCTAGAGAAC
	Reverse	ACACATTCGCCTAGAGAAC
YTHDF3	Forward	CAAGTGCAGTCTCAACAGCCA
	Reverse	CAGCACTGGATGCACCTCT

Supplementary Table S5

Antibodies used in the this study

**Product**	**Source**	**No. of Catalogue**
Anti-TRIM50	Novus	NBP2-85994
Anti-TRIM50	Novus	NBP2-81988
Anti-PGK1	Thermo Fisher	MA5-37712
Anti-CD68	Thermo Fisher	14-0681-82
Anti-CD68-FITC	Becton Dickinson and Company	#562117
Anti-CD163	Thermo Fisher	MA5-11458
Anti-CD163-APC	Thermo Fisher	17-1639-42
Anti-N-cadherin	Thermo Fisher	PA5-19486
Anti-E-cadherin	Thermo Fisher	MA5-15711
Anti-Vimentin	Thermo Fisher	14-9897-82
Anti-snail	Thermo Fisher	14-9859-82
Anti-GAPDH	Abcam	ab8245
Anti-β-Tubulin	Abcam	ab179513
Anti-Ki67	Abcam	ab15580
Anti-Smad3	Abcam	ab40854
Anti-p-Smad3	Abcam	ab52903
Anti-Smad2	Abcam	ab40855
Anti-p-Smad2	Abcam	ab280888
Anti-JAK2	Abcam	ab108596
Anti-p-JAK2	Abcam	ab32101
Anti-STAT3	Abcam	ab68153
Anti-p-STAT3	Abcam	ab267373
Anti-METTL3	Cell Signaling Technology	#86132
Anti-YTHDF2	Cell Signaling Technology	#71283
Anti-Flag	Sigma-Aldrich	F3165
Anti-HA	Sigma-Aldrich	H3663
IgG	Abcam	ab172730
anti-rabbit IgG-HRP	Proteintech	SA00001-15
anti-mouse IgG-HRP	Proteintech	SA00001-1
Goat Anti-Mouse IgG H&L (Alexa Fluor® 647)	Abcam	ab150115
Goat Anti-Rabbit IgG H&L (Alexa Fluor® 594)	Abcam	ab150080
Goat Anti-Rat IgG H&L (Alexa Fluor® 488)	Abcam	ab150165

Supplementary Table S6

The overexpression plasmids used in this study

Plasmid	Vector	Supplier (Catalog No.)
Flag-TRIM50	pEnCMV-TRIM50-3XFLAG	Dongxuan Bio
HA-PGK1	pEnCMV-PGK1-3XFLAG	Dongxuan Bio
Ubiquitin-HA	pRK5-HA-Ubiquitin-WT	Miaoling Bio (P1761)
HA-Ub-K48	pCMV-HA-UB-K48	Miaoling Bio (P31802)
HA-Ub-K63	pCMV-HA-UB-K63	Miaoling Bio (P31800)
HA-Ub-K48R	pCMV-HA-Ub-K48R	Miaoling Bio (P8355)
HA-Ub-K63R	pCMV-HA-Ub-K63R	Miaoling Bio (P0855)

Supplementary Table S7

The siRNAs used in the experiments

siRNA		Sequence 5'-3'
SiRNA	Sense	UUCUCCGAACGUGUCACGUTT
	Antisense	ACGUGACACGUUCGGAGAATT
SiTRIM50#1	Sense	GGCCCUUAGAAGGCGCAUUTT
	Antisense	AAUGCGCCUUCUAAGGGCCTT
SiTRIM50#2	Sense	CCGGGUGUACGAAGCCUUUTT
	Antisense	AAAGGCUUCGUACACCCGGTT
SiMETTL3#1	Sense	GGAGAUGGAGACUUCUUUATT
	Antisense	UUAAAGAAGUCUCCAUCUCTT
SiMETTL3#2	Sense	GCCAAUAUCAAGAAACUUUTT
	Antisense	AAAGUUUCUUGAUAUUGGCTT
SiMETTL14#1	Sense	GAAGGACUCUCCAGAACUATT
	Antisense	UAGUUCUGGAGAGUCCUUCTT
SiMETTL14#2	Sense	GCACAAUACUUGCUUGAAATT
	Antisense	UUUCAAGCAAGUAUUGUGCTT
SiWTAP#1	Sense	GAAGACAGCUGGACUAUUATT
	Antisense	UAAUAUAGUCCAGCUGUCUTT
SiWTAP#2	Sense	GCCAGAUUAGGACUUGAAATT
	Antisense	UUUCAAGUCCUAAUCUGGCTT
SiRBM15#1	Sense	GAAGAUUGGUUCUCAGAAATT
	Antisense	UUUCUGAGAACCAAUCUUCTT
SiRBM15#2	Sense	GCCAUGUUACUUACAAUGATT
	Antisense	UCAUUGUAAGUAACAUUGGCTT
SiFTO#1	Sense	GCAGUGCCUUGGUGUUCAATT
	Antisense	UUGAACACCAAGGCACUGCTT
SiFTO#2	Sense	GGUGAAGAUUCCUGGAAUAATT
	Antisense	UUUAUUCCAGGAAUCUCCACTT
SiALKBH5#1	Sense	GCGAGAUACUGAACAGUAATT
	Antisense	UUACUGUUCAGUAUCUCGCTT
SiALKBH5#2	Sense	GCAAGAAUGUGGCUCGCUATT
	Antisense	UAGCGAGCCACAUCUUCUGCTT
SiIGF2BP1#1	Sense	GCAGGAAUUGGUGAAUCAATT
	Antisense	UUGAUUCACCAAUUCCUGCTT
SiIGF2BP1#2	Sense	GCCAUUGAGUCGACUGAAATT
	Antisense	UUUCAGUCGACUCAAUGGCTT
SiIGF2BP2#1	Sense	GCAGGAACGGUGAAUUCUATT
	Antisense	UAGAAUUCACCGUUCCUGCTT
SiIGF2BP2#2	Sense	GCAUGGAGCUUCAUCUGAATT
	Antisense	UUCAGAUGAAGCUCCAUGCTT
SiIGF2BP3#1	Sense	GCACCGUUCUACUGGACAAATT

	Antisense	UUUGUCCAGUAGAACGGUGCTT
SiIGF2BP3#2	Sense	GGAAGGAGAUUGGCAGAUUATT
	Antisense	UAAUCUGCCAUCUCCUUCCTT
SiYTHDC2#1	Sense	GCCAAUGGCUUGUACUACUTT
	Antisense	AGUAGUACAAGCCAUU GGCTT
SiYTHDC2#2	Sense	GCAGAUGGCUAGAAUCCUATT
	Antisense	UAGGAUUCUAGCCAUCUGCTT
SiYTHDF2#1	Sense	GCCAAUGGCUUGUACUACUTT
	Antisense	AGUAGUACAAGCCAUUGGCTT
SiYTHDF2#2	Sense	GCAAGUGUCUUCACCAAAATT
	Antisense	UUUUGGUGAAGACACUUGCTT
SiYTHDF3#1	Sense	GCAGGAAUCUGACAGAAUATT
	Antisense	UAUUCUGUCAGAUUCCUGCTT
SiYTHDF3#2	Sense	GCCAUUGUCUACAGACUUCTT
	Antisense	GAAGUCUGUAGACAAUGGCTT

Supplementary Table S8

Reagents used in the present study

**Product**	**Source**	**No. of Catalogue**
Cycloheximide	MedChemexpress	HY-12320
MG132	MedChemexpress	HY-13259
Chloroquine	MedChemexpress	HY-17589
Galloflavin	MedChemexpress	HY-W040118
5-Aza-2'-deoxycytidine	MedChemexpress	HY-10586
Cycloleucine	MedChemexpress	HY-30008
PMA	Sigma-Aldrich	P1585
IL-4	PeproTech	200-04
IL-13	PeproTech	200-13
Lactate	Sigma-Aldrich	1614308

Supplementary methods

2.6 Construction and transfection of plasmids and lentiviral vectors

Target-specific small interfering RNAs (siRNAs) targeting TRIM50 and control siRNA were constructed by RiboBio (Guangzhou, China). Lentiviral vectors containing TRIM50 siRNA hairpin sequences and their complementary antisense sequences were constructed by GenePharma. Lentiviral vectors containing PGK1 siRNA hairpin sequences and their complementary antisense sequences were also constructed by GenePharma. Flag-TRIM50 and HA-PGK1 were obtained from Dongxuan Bio (Suzhou, China). Different TRIM50 domain deletion mutants were generated using the KOD-Plus Mutagenesis Kit (Toyobo, Osaka, Japan) according to the provided protocol. Different PGK1 ubiquitination site deletion mutants were obtained using the QuikChange site-directed mutagenesis kit (Agilent Technologies, Santa Clara, California, USA). HA-UB, HA-UB-K48, HA-UB-K48R, HA-UB-K63, and HA-UB-K63R plasmids were constructed by Miaoling Bio (Wuhan, China). All constructs were confirmed by DNA sequencing. Plasmids were transfected into cells using Lipofectamine 3000 (Invitrogen, USA) following the manufacturer's instructions. GC cells were transfected with either control lentivirus (pLKO.1 or pCMV) or lentivirus containing TRIM50 or PGK1 overexpression or knockdown constructs according to the manufacturer’s instructions. The GFP+HGC-27 and GFP+MKN-45 cells were generated through transfection with a control lentivirus carrying GFP gene (Vigene Biosciences, Shandong, China). It is noteworthy that all lentiviral constructs used in this study were engineered to include the Firefly luciferase gene. Stable transfectants were selected using puromycin hydrochloride (MedChemExpress, Shanghai, China). The transfection efficiency was validated by qRT–PCR or western blotting. The plasmids and siRNAs used in the experiments are shown in Supplementary Table S6 and Table S7.

2.7 Differentiation of THP-1 Cells into Macrophages

For differentiation into macrophages, 1 x 10^6 THP-1 cells were seeded in 100 mm culture dishes and treated with 100 ng/mL phorbol 12-myristate 13-acetate (PMA) (Sigma-Aldrich) to induce differentiation. The cells were treated with PMA for 48 hours, after which the medium was replaced with macrophage differentiation medium (Gibco, Thermo Fisher Scientific) without PMA. The cells were then continuously cultivated in a 37 °C incubator with 5% CO2 to facilitate maturation. Throughout this process, it was important to monitor the cells for morphological changes that indicate macrophage differentiation. The differentiation status could be confirmed by assessing the expression of macrophage-specific markers using flow cytometry or immunofluorescence. Reagents used in the experiments are shown in Supplementary Table S8.

2.8 M2 Polarization of THP-1-Derived Macrophages and Co-culture of Macrophages with TRIM50-intervened GC Cells

To polarize the differentiated macrophages into the M2 phenotype, the macrophage differentiation medium was supplemented with 20 ng/mL interleukin-4 (IL-4) (PeproTech) and 10 ng/mL interleukin-13 (IL-13) (PeproTech) for an additional 48 hours.

In parallel, to study the effect of lactate on M2 polarization, M0 macrophages differentiated from THP-1 were treated with lactate (Sigma-Aldrich) at a final concentration of 10 mM for 48 hours. This treatment was performed in the presence of the GC cell supernatant to investigate the synergistic effects of lactate on M2 polarization.

For experiments involving co-culture of macrophages with GC cells, GC cells after TRIM50 expression intervention were seeded in transwell inserts (Corning) with a porous membrane that allowed for the exchange of soluble factors but prevented direct cell-cell contact. M0 macrophages differentiated from THP-1 were plated in the lower chamber, and the co-culture was maintained for an additional 24-48 hours to assess the role of TRIM50 expression in GC cells on macrophage M2 polarisation. The M2 macrophages and supernatants were then harvested for co-culture experiments or further analysis. Reagents used in the experiments are shown in Supplementary Table S8.

2.9 Colony Formation Assay

HGC-27, MKN-45, and AGS cells (with 500 cells per well) were cultured in a 6-well plate for a duration of two weeks, with medium changes performed every 3-5 days. On the 14th day, the colonies were fixed using a 4% paraformaldehyde solution for 15 minutes, followed by staining with a 0.1% crystal violet solution for 30 minutes. The proliferative capacity of GC cells was primarily evaluated based on the quantity and size of the clones. All experiments were performed in triplicate.

2.10 Cell Counting Kit-8 (CCK-8) Assay

GC cells were seeded into a 96-well plate with 100 μL of culture medium per well (2×10^3 cells per well). Subsequently, CCK-8 solution (Dojindo, Kumamoto, Japan) was added to each well (10 μl per well), followed by incubation at 37 °C for 2 hours. Afterward, cell viability was monitored for 5 days using a microplate reader (BioTek, Winooski, VT, USA) at a wavelength of 450 nm. All experiments were performed in triplicate.

2.11 5-Ethynyl-2'-deoxyuridine (EdU) Assay

The EdU assay was performed following the instructions provided by the manufacturer of the Cell-light TM EdU DNA Cell Proliferation Kit (RiboBio, Guangzhou, China). In brief, MKN-45 and HGC-27 cells (1×10^3 cells per well) were seeded into a 96-well plate. On the following day, GC cells were incubated with 50 μM EdU for 2 hours. Subsequently, the cells were fixed with 4% paraformaldehyde and stained using the Apollo Dye Solution. After staining the GC cell nuclei

with Hoechst 33342, image acquisition was conducted using an Olympus microscope (Olympus, Tokyo, Japan). All experiments were performed in triplicate.

2.12 Wound Healing Assay

GC cells (HGC-27 and MKN-45) for both the intervention and control groups were inoculated into culture dishes. After the cells reached confluence, the monolayer was wounded with a 200 µL pipette tip, and detached cells were removed with PBS. The cells were then cultured in a serum-free medium to inhibit proliferation. Images were taken at 0 and 24 h, and the area of the wound was measured using ImageJ software. All experiments were performed in triplicate.

2.13 Transwell Assay

To assess cell invasion, 24-well BioCoat Matrigel Invasion Chambers (BD, Biosciences, Franklin Lakes, USA) were utilized following the manufacturer's protocol. The upper chamber was seeded with 3×10^4 cells in serum-free culture medium, while the lower chamber was filled with medium containing 10% FBS. The cells were cultured for 24 hours. Afterward, the cells on the upper surface of the filter were gently wiped off, and the cells on the lower surface were stained with 0.1% crystal violet (Sigma) for 30 minutes. Subsequently, the number of invading cells was counted under a microscope. Migration assay was performed using the same procedure, except that the filter was not pre-coated with Matrigel. All experiments were performed in triplicate.

2.14 Coimmunoprecipitation (Co-IP)

Co-IP was performed to determine the interaction between TRIM50 and PGK1 proteins. Cells were collected and lysed with 0.6 ml of lysis buffer plus protease inhibitors (Roche Applied Science) for 40 min on ice. After centrifugation at 12,000 g for 15 min, the lysates were immunoprecipitated with specific antibody overnight for 1 h at 4 °C, and 30ul protein A agarose beads (Invitrogen) or protein G agarose beads (Santa Cruz) were washed and then added for an additional 3 h. Thereafter, the precipitants were washed four times with lysis buffer, and the protein A-agarose-antigen-antibody complexes were boiled with loading buffer for 3 min and analyzed by SDS–PAGE and Western blotting. IP with a rabbit IgG isotype was conducted as a negative control.

2.15 Mass spectrometry analysis

The Pierce™ Classic Magnetic IP/Co-IP Kit (Thermo Fisher) was used (according to the manufacturer's instructions) to examine proteins coupled to TRIM50. Cells (1×10^7) were washed in PBS twice and then 500 µL IP lysis buffer (containing 1× Protease Inhibitor Cocktail) was added to lyse cells. One tenth of the supernatant was saved as the input. To the remaining supernatant, Flag antibody magnetic bead complex was added and then samples were incubated at

4°C overnight. Beads were washed five times with buffer containing 10 μg/mL RNAse A before being eluted by 100 μL elution buffer. Protein samples (20 μL) were boiled in LDS buffer for western blotting, and 80μL protein samples were analyzed by label-free LC/MS.

2.16 Methylated RNA–immunoprecipitation (MeRIP)

The MeRIP assay was performed according to the manufacturer's instructions of Magna MeRIP™ m6A Kit (17-10499; Millipore, Billerica, Massachusetts, USA). In brief, RNA was chemically fragmented into 100 nucleotides or smaller fragments, followed by magnetic immunoprecipitation with a monoclonal antibody (1:1000, MABE1006, Millipore) toward m6A. After washing with IP buffer, the RNA was eluted and precipitated with ethanol. Then, the isolated RNA fragments could be subjected to qRT–PCR and Agarose gel electrophoresis analysis, which were normalized to input.

2.17 Lactate, glucose, and ATP assays

For lactate measurement, we utilized a Lactate Assay Kit (K627, BioVision) to determine lactate concentration in whole-cell lysates according to the manufacturer's instructions.

For glucose uptake measurement, the indicated cells were incubated with 100 μM 2-NBDG (11046, Cayman) for 30 min, followed by washing with chilled PBS. Subsequently, FL-1 fluorescence was measured and was recorded according to the manufacturer's instructions.

For ATP measurement, we used an ATP Assay Kit (K354, BioVision) to detect intracellular ATP in whole-cell extracts by monitoring luciferase activity.

All experiments were performed in triplicate.

2.18 Measurement of ECAR and OCR

A Seahorse XF96 extracellular flux analyzer (Seahorse Biosciences, USA) was utilized to determine the extracellular acidification rate (ECAR) and oxygen consumption rate (OCR). The Seahorse XF Glycolysis Stress Test Kit (103020, Agilent) and Seahorse XF Cell Mito Stress Test Kit (103010, Agilent) were used to quantitatively measure the ECAR and OCR in various experimental groups. Specifically, a working solution consisting of 175 mmol of Seahorse buffer and 25 mmol of glucose (10 μ/l), oligomycin (1 mmol/l), and 2-deoxyglucose (100 mmol/l) was added to the probe plate for the measurement of ECAR. Alternatively, an automatic injection of 175 mmol/l Seahorse buffer into the analyzer, containing 25 mmol/l of oligomycin (1 μ/l), carbonyl cyanide p-trifluoromethoxyphenylhydrazone (2 μ/l), and antimycin A/rotenone (0.5 mmol/l) was used for the measurement of OCR. Data analysis was conducted using the Seahorse XF-96 Wave software, with ECAR recorded in mpH/min and OCR recorded in pmol/min. All experiments were performed in triplicate.

2.19 Measurement of intracellular ROS levels

To quantify the intracellular levels of reactive oxygen species (ROS) in each cell group, a Reactive Oxygen Species Assay Kit (S0033S, Beyotime) was used according to the manufacturer's instructions. The frequency of fluorescence-positive cells was quantified using flow cytometry with the assistance of CellQuest software. All experiments were performed in triplicate.

2.20 Measurement of the NAD+/NADH ratio

The levels of intracellular NAD+/NADH in each cell group were quantified using an NAD/NADH-Glo Assay Kit (Promega, G9072). The NAD+ and NADH levels were measured using a spectrophotometer (Tecan Infinite, M200Pro) according to the instructions provided in the Promega G9072 technical manual. All experiments were performed in triplicate.

2.21 Flow cytometry

Macrophages were obtained and processed into a single-cell suspension, followed by incubation with antibodies (anti-CD68-FITC, anti-CD163-APC) at 4 °C for 1 h. Subsequently, the cells were washed twice with 4 ml of flow buffer, centrifuged, and resuspended in 0.5 ml of flow buffer for analysis. Flow cytometry was performed using a Cytoflex flow cytometer (Beckman Coulter, USA). Data analysis for flow cytometry was conducted using FlowJo software (FlowJo, USA). All experiments were performed in triplicate.

2.22 In vitro ubiquitination assay

Cells were cultured until they reached 80%-90% confluency in a culture dish. HA-Ub was transfected into the cells using Lipofectamine 3000. Cells were then transfected with either a TRIM50 overexpression, TRIM50 knockdown, or empty vector plasmid. After 36 h, MG132 (final concentration: 10 μM) was added to the cells and incubated for 6 h. RIPA lysis buffer was prepared and used to extract protein substrates for immunoprecipitation. Magnetic beads cross-linked with anti-PGK1 antibodies were added, and immunoprecipitation was performed to pull down the HA-tagged protein. The samples were incubated overnight at 4 °C with rotation. The ubiquitination level of PGK1 was detected by Western blotting with an anti-HA antibody. Reagents used in the experiments are shown in Supplementary Table S8.

2.23 Cytokine Measurement

The supernatant of the macrophage culture model was analyzed for inflammatory cytokines associated with invasion, including TGF-β, MCP-1, IP-10, IL-6, VEGF, G-CSF, IL-15, IL-1β, IL-10, and IL-12, using the human cytokine antibody array membrane-based ELISA kit provided by Huayn Biotech (Shanghai, China). The results were expressed in picograms per milliliter (pg/ml). All experiments were performed in triplicate.

2.24 Immunofluorescence (IF) Staining

(1) Cellular Immunofluorescence Staining

Cells were gently washed with PBS and subsequently fixed in a chilled acetone-methanol mixture (1:1) for a duration of 5 minutes. Following three thorough rinses with PBS, the cells were incubated at room temperature in a blocking buffer consisting of PBS supplemented with 3% donkey serum and 0.3% Triton X-100 at a pH of 7.4 for a period of 2 hours. Subsequently, the cells were adorned with primary antibodies and allowed to incubate overnight at a temperature of 4 °C in the presence of 5% normal goat serum in PBS. Secondary antibodies, conjugated with either Alexa Fluor 594 or 488, were utilized to visualize the primary antibodies, while 4,6-diamidino-2-phenylindole (DAPI) was employed for cellular counterstaining. Visualization and imaging of the samples were accomplished using the advanced Stellaris STED laser confocal microscope (Olympus, Japan).

(2) Tissue Immunofluorescence Staining

Tumor tissue or pituitary tissue specimens were meticulously fixed in a 4% paraformaldehyde solution for a duration of 24 hours, followed by embedding in paraffin. Paraffin blocks were skillfully sectioned into slices with a thickness of 5 μm. After three successive washes with PBS, the sections were incubated at room temperature in a blocking solution for a period of 2 hours. The samples were adorned with primary antibodies against TRIM50, CD68, and CD163 and allowed to incubate overnight at a temperature of 4°C in an antibody reaction buffer comprised of 1% bovine serum albumin (BSA) and 0.3% Triton X-100 in PBS. Subsequently, the samples were incubated with secondary antibodies conjugated with Alexa Fluor 674, 488, or 594. Following a 1-hour incubation at room temperature, nuclear staining was performed using DAPI. The cutting-edge Thunder Imager fast high-resolution inverted fluorescence imaging system (Leica Microsystems, Germany) was utilized for visualization and capturing of the images.

2.25 Immunohistochemistry (IHC) analysis and hematoxylin-eosin (HE) staining

The tumor tissues were fixed in a 4% paraformaldehyde solution after excision and subsequently embedded in paraffin. For immunohistochemistry (IHC), tissue sections with a thickness of 5 μm were incubated with primary antibodies at 4 °C overnight, followed by incubation with secondary antibodies at room temperature for 1 h. Following staining with a chromogen (Servicebio, Wuhan, China), the tissue sections were imaged for examination. Hematoxylin-eosin (HE) staining was performed using a Beyotime Hematoxylin-Eosin staining kit (Shanghai, China), directly staining the cell nuclei and cytoplasm. Details regarding the primary and secondary antibodies used in IHC and HE staining can be found in Supplementary Table S5.

2.26 Immunohistochemical scoring

Immunohistochemical scoring was performed using a scoring system that combines cell staining intensity and the extent of positive cell staining on tissue sections. Cell Staining Intensity: Grade 0: No positive staining (negative) scores 0 points. Grade 1: Pale yellow staining (weakly positive) scores 1 point. Grade 2: Brownish-yellow staining (positive) scores 2 points. Grade 3: Brownish-brown staining (strong positive) scores 3 points. Percentage of Positive Cells: Grade 1: ≤ 25% positive cells score 1 point. Grade 2: 26%-50% positive cells score 2 points. Grade 3: 51%-75% positive cells score 3 points. Grade 4: > 75% positive cells score 4 points. The final score is calculated by multiplying the scores from both criteria. Five pathologists evaluated the immunohistochemistry slides according to the aforementioned criteria, and the average score was considered the final score.

2.27 Animal experiments

The animal experimental protocol involved in this study was reviewed and approved by the Animal Ethics Committee of Nanjing Medical University.

For the in vivo tumor growth study, HGC-27 and MKN-45 cells were transfected with lentiviral vectors. Twenty-four BALB/c nude mice (4 weeks old) were randomly divided into four groups. Subsequently, HGC-27 and MKN-45 cells (1×10^7 cells dissolved in 150 μL PBS) were subcutaneously injected into the axillary region of each mouse's forelimb. The volume of xenograft tumors was measured weekly using the formula $V = \text{length} \times \text{width}^2 \times 0.5$. After 4 weeks, the mice were euthanized, and the subcutaneous tumors were harvested, weighed, and stained using antibodies against Ki-67, TRIM50, and PGK1.

In the mouse metastasis models, HGC-27 and MKN-45 cells transfected with lentiviral vectors were cocultured with THP-1-induced M0 macrophages for 24 h. The supernatant of the cocultured macrophages was collected. A mixture of GC cells (1×10^6) and the supernatant of macrophages (100 μL) was injected into the tail vein and spleen of BALB/c nude mice (5 weeks old). Subsequently, every other day, 100 μL of macrophage supernatant was injected into the mice via the tail vein and spleen to establish a mouse model of GC lung and liver metastasis. After 4 weeks, lung and liver metastases in these mice were examined using bioluminescence imaging. Following anesthesia with 2% isoflurane, a stock solution of D-luciferin sodium salt (150 mg/ml) was injected into each mouse’s peritoneal cavity. Subsequently, the anesthetized mice were placed on the IVIS SpectrumD Xenogen imaging system (Caliper Life Sciences, Hopkinton, Massachusetts, USA) for measurement of the values of bioluminescence imaging signals. After imaging, all nude mice were euthanized, and their lungs and livers were harvested for HE staining to further evaluate lung and liver metastases.
